# Validation of prognostic models predicting mortality or ICU admission in patients with COVID-19 in low- and middle-income countries: a global individual participant data meta-analysis

**DOI:** 10.1186/s41512-024-00181-5

**Published:** 2024-12-19

**Authors:** Johanna A. A. Damen, Banafsheh Arshi, Maarten van Smeden, Silvia Bertagnolio, Janet V. Diaz, Ronaldo Silva, Soe Soe Thwin, Laure Wynants, Karel G. M. Moons

**Affiliations:** 1 Julius Center for Health Sciences and Primary Care, University Medical Center Utrecht, Utrecht University, Utrecht, 3508 GA the Netherlands; 2 Department of Epidemiology, CAPHRI Care and Public Health Research Institute, Maastricht University, Maastricht, the Netherlands; 3 World Health Organization, Geneva, Switzerland; 4 Department of Development and Regeneration, KU Leuven, Louvain, Belgium

## Abstract

**Background:**

We evaluated the performance of prognostic models for predicting mortality or ICU admission in hospitalized patients with COVID-19 in the World Health Organization (WHO) Global Clinical Platform, a repository of individual-level clinical data of patients hospitalized with COVID-19, including in low- and middle-income countries (LMICs).

**Methods:**

We identified eligible multivariable prognostic models for predicting overall mortality and ICU admission during hospital stay in patients with confirmed or suspected COVID-19 from a living review of COVID-19 prediction models. These models were evaluated using data contributed to the WHO Global Clinical Platform for COVID-19 from nine LMICs (Burkina Faso, Cameroon, Democratic Republic of Congo, Guinea, India, Niger, Nigeria, Zambia, and Zimbabwe). Model performance was assessed in terms of discrimination and calibration.

**Results:**

Out of 144 eligible models, 140 were excluded due to a high risk of bias, predictors unavailable in LIMCs, or insufficient model description. Among 11,338 participants, the remaining models showed good discrimination for predicting in-hospital mortality (3 models), with areas under the curve (AUCs) ranging between 0.76 (95% *CI* 0.71–0.81) and 0.84 (95% *CI* 0.77–0.89). An AUC of 0.74 (95% *CI* 0.70–0.78) was found for predicting ICU admission risk (one model). All models showed signs of miscalibration and overfitting, with extensive heterogeneity between countries.

**Conclusions:**

Among the available COVID-19 prognostic models, only a few could be validated on data collected from LMICs, mainly due to limited predictor availability. Despite their discriminative ability, selected models for mortality prediction or ICU admission showed varying and suboptimal calibration.

**Supplementary Information:**

The online version contains supplementary material available at 10.1186/s41512-024-00181-5.

## Introduction

The ongoing COVID-19 pandemic remains a global health threat, with more than six million deaths attributed to this infection reported worldwide to the World Health Organization (WHO) by January 2023 [1]. Despite all efforts to limit its spread and control the disease, COVID-19 has remained a global crisis, and SARS-CoV-2 is cautioned to persist as an endemic virus with seasonal outbreaks [2, 3]. The unpredictable changing potential of SARS-CoV-2 highlights the need for strategies to respond to this virus in all parts of the world [2].

Assessing the risk of adverse outcomes, and mortality in particular, among patients diagnosed with COVID-19, using prognostic models can guide healthcare and public health work force in decision-making and efficient allocation and management of resources and hospital infrastructures. Of the numerous published COVID-19 prognostic models, most lack complete reporting and have a high risk of bias [4, 5]. Most of these models have not been validated using external data. Therefore, information on the performance, stability, and transportability of these prognostic models across populations and settings is currently limited.

Low- and middle-income countries (LMICs) have been severely burdened by COVID-19 [6]. COVID-19 mortality rates have been substantially higher in developing countries than in high-income countries (HICs) [6, 7]. Disparities in mortality have been attributed to, for example, variation in demographics between countries, higher prevalence of comorbidities [7], or other environmental factors in LIMCs [8]. LMICs also have different treatment modalities and disease management, fewer vaccinations [5], lack of resources such as ICUs or laboratory capacity [6, 9], and understaffed healthcare systems. COVID-19 prognostic models — mainly developed in HICs — may therefore perform worse when applied in low-resource settings than their originally reported performance indicates. It is of interest to evaluate the performance and applicability of these existing prediction models in LIMCs [5, 9].

In this study, we identified existing models for predicting mortality or ICU admission in hospitalized patients with COVID-19 applicable in LMICs to assess their performance in a large global dataset from the WHO Global Clinical Platform [10] using an individual participant data meta-analysis (IPD-MA) approach. To our knowledge, the WHO Clinical Platform is the largest COVID-19 clinical dataset; as of November 2022, it included over 1 million cases from 64 countries. For this analysis, we used a dataset from 10 LMICs and chose hard outcomes such as mortality and ICU admission, which carry limited heterogeneity between countries in definitions and measurement.

## Methods

This study is reported in accordance with transparent reporting of a multivariable prediction model for individual prognosis or diagnosis (TRIPOD) guidance (www.tripod-statement.org) [11, 12].

### Identifying COVID-19 prognostic prediction models

We used the fourth update (search date 17 February 2021) of the living systematic review of prediction models for COVID-19 to identify existing multivariable prognostic models for validation [4]. In short, this systematic review included diagnostic and prognostic multivariable models or scoring systems developed or validated to predict any COVID-19-related outcome. The quality of the prediction models was assessed using the Prediction Model Risk-Of-Bias Assessment Tool (PROBAST; www.probast.org) [13, 14].

Models eligible for inclusion in the current IPD-MA were prognostic models developed for hospitalized patients with confirmed or suspected COVID-19 predicting outcomes uniformly available in the LMIC dataset (mortality, ICU admission, or a composite of the two). Studies validating existing models (not originally developed for COVID-19 patients) were not eligible. From the eligible models, we excluded those with a high risk of bias for the PROBAST domains of participants or predictors. We also excluded models consisting of predictors unavailable in our LMIC dataset (e.g., blood biomarkers) or models using predictors with more than 80% missing values in our dataset. Models that were not fully presented in the published article and for which no code or information was provided upon e-mail request were excluded. The remaining models were included for evaluation of their predictive performance in LMICs. For each included model, we extracted predictor definitions, outcome definitions (including time horizons), and final model parameters from original publications. In case prognostic models were only presented as a nomogram, we used previously developed methods to obtain the regression formula [15].

### Validation of selected models in LMICs

Selected prognostic models were evaluated using data from the large WHO Global Clinical Platform database for COVID-19 based on the standardized WHO Case Report Form (CRF). The platform is intended to provide a standardized approach and platform to collect clinical data of COVID-19 patients worldwide to characterize the natural history of the disease, identify predictors for severe illness, and describe treatment interventions. The WHO CRF is divided into four modules, the first to be completed on the first day of inpatient admission to the healthcare facility, the second to be completed on ICU admission or ICU transfer, the third to be completed at the time of discharge or death, and the fourth to be completed if currently pregnant or less than 22 days from pregnancy outcome. The CRF contains a standardized set of variables, including country, demographics, underlying conditions, vital signs, anthropometric measurements, clinical symptoms, medications use, laboratory testing, oxygen supplementation, complications occurring due to COVID-19, and clinical outcomes (discharge, in-hospital death, transfer to another facility, or remaining hospitalized at the time of data collection). An overview of all relevant predictor and outcome definitions is provided in the supplement.

The WHO Clinical Platform contains anonymized individual-level data from patients worldwide admitted to a healthcare facility with suspected or confirmed COVID-19. We included patients admitted between 3 January 2020 and 30 September 2021 from 10 LMICs (according to the World Bank definition [16]): Burkina Faso, Cameroon, Democratic Republic of Congo, Ghana, Guinea, India, Niger, Nigeria, Zambia, and Zimbabwe. In a later stage, participants from Ghana were excluded as their clinical data lacked information on mortality outcomes.

### Statistical analysis

Distributions of predictor variables included in the selected models and the collected outcomes in the LMICs were described using median and interquartile range (IQR) for continuous variables and absolute numbers and percentages (%) for categorical variables.

The presence of missing data in the predictor variables and outcomes was explored, including the presence of sporadically missing data and systematically missing data (i.e., missingness of all individuals on a predictor variable from one or more LMICs). Systematic and sporadic missing data were imputed 50 times using joint modeling multiple imputations accounting for our data’s multilevel structure [17]. The imputation model included the variables of the validated prognostic models and the outcomes predicted by these models (mortality and ICU admission). More details on the imputation models can be found in the supplement.

For each included prognostic model, we calculated the predicted risk of in-hospital mortality or ICU admission (depending on the indented outcome of the model) in every imputed dataset. Details of model equations can be found in the supplement. Subsequently, for individual countries, performance was calculated in terms of discrimination (AUC) and calibration (ratio of observed over the expected number of events (OE ratio), calibration slope, calibration-in-the-large). Estimates of performance measures were then pooled across imputations using Rubin’s rules and visualized in forest plots. As a sensitivity analysis, estimates of performance measures were also combined using medians [18]. Performance measures for each evaluated model were subsequently pooled across the LMICs using restricted maximum likelihood estimation and applying the Hartung-Knapp-Sidik-Jonkman method for calculating 95% CIs [19]. A total of 95% prediction intervals (PIs) were also constructed [20]. These indicate likely values for performance when the models are applied in a new but similar country. We performed pooling on the logit scale for the AUC and the log scale for the OE ratio. We assessed the calibration of models in terms of the agreement between the observed and estimated probabilities by plotting the observed probabilities of outcomes against their estimated probabilities from each model, incorporating a smoothed nonlinear curve generated using a loess smoother in the total population and by country. We did not draw calibration plots for countries with fewer than 50 outcome events. Calibration plots across imputations were drawn for stacked imputed datasets [21].

All analyses were performed in R version 4.1.3 using packages mitml, pROC, metamisc, and metafor.

#### Patient and public involvement

Patients and public were not involved in this study.

## Results

### Identifying COVID-19 prognostic prediction models

**Fig. 1:**
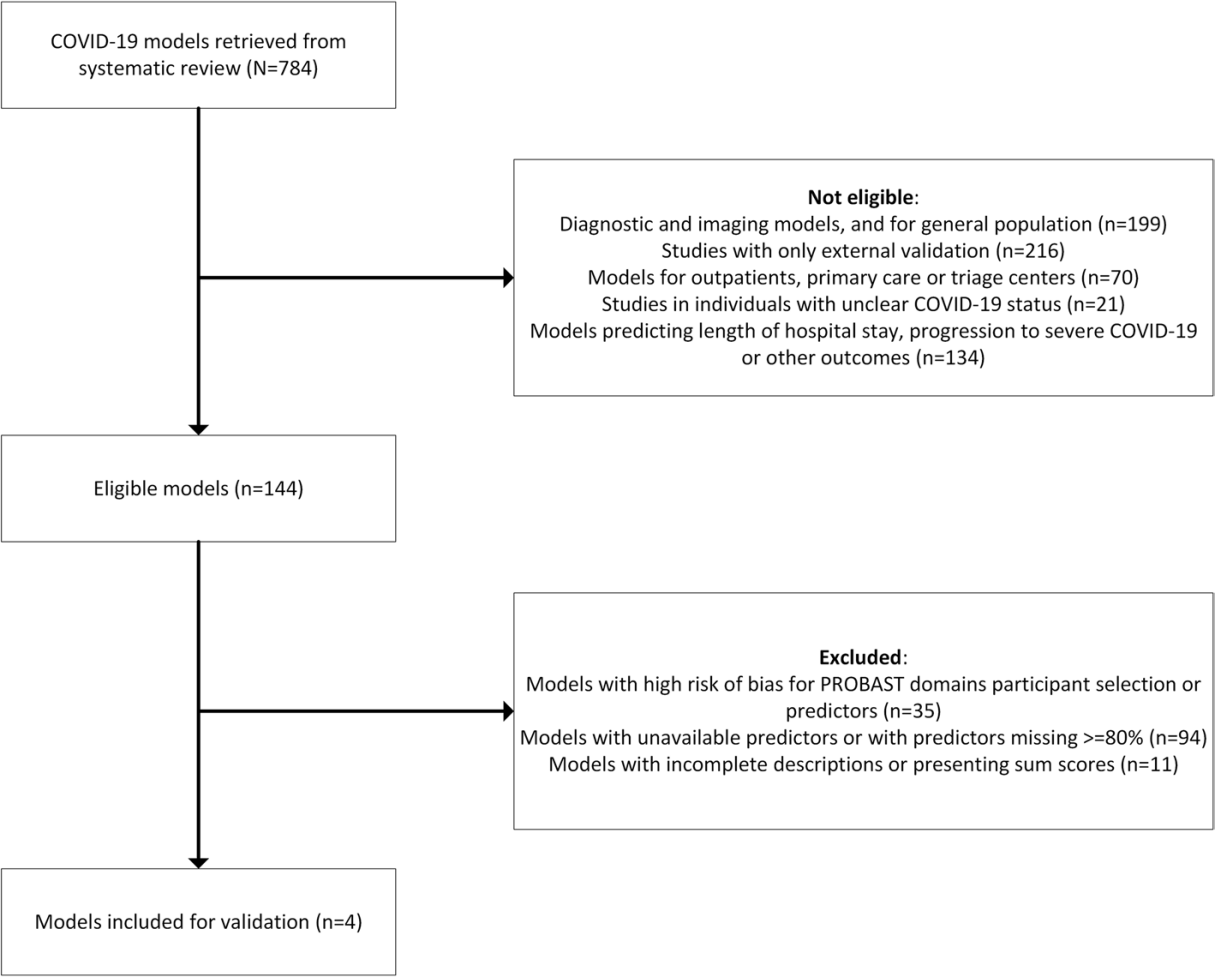
Flow chart for model selection in the study

The fourth update of the living review on prediction models for COVID-19 described 784 candidate prediction models [4]. Models eligible for this study were prognostic models developed for hospitalized patients with confirmed or suspected COVID-19 for outcomes in the available dataset, including mortality and ICU admission or a composite outcome of the two (*n* = 144) (Fig. 1). An overview of these eligible models and their risk of bias is available in the Supplemental Table 1.

We excluded models with a high risk of bias for the PROBAST domains participant selection or predictors (*n* = 35). We excluded an additional 94 models due to the unavailability of predictors or predictors having more than 80% missing in the LMIC data (such as imaging or blood values). Eleven models were excluded because the full model or an alternative presentation that allowed the calculation of predicted risks in new patients was not described nor provided by the authors after a request by e-mail. Four prediction models were included in the external validation [22–25]. Three of these models (developed by Berzuini et al., Wang et al., and Zhang et al.) predict the risk of in-hospital mortality, while the model by Zhou et al. was developed to predict the risk of ICU admission. Further details of selected models for validation are presented in Table 1.

**Table 1:** Overview of prognostic models selected in the study

Authors	Country	Study dates	Number of events/participants for model development	Outcome	Predictors in the model	Prediction horizon	Model presentation	Predictive performance in development study	Risk of bias: participants	Risk of bias: predictors	Risk of bias: outcome	Risk of bias: analysis
Berzuini et al	UK	March 11th until April 17th, 2020	110/392	Mortality in hospital	Age, O^2^ saturation, respiratory rate, smoking	21 days	Logistic regression	AUC^a^: 0.73	Unclear	Low	Low	High
Wang et al	China	January 7th until February 20th, 2020	19/296 (14/44)c	Mortality in hospital	Age, hypertension, coronary heart disease	Unclear	Nomogram	C-statistic (95% CI)^b^: 0.83 (0.68 to 0.93) Sensitivity^c^: 64% Sensitivity^c^: 93%	Low	Low	Low	High
Zhang et al	China	February 1st until 23rd, 2020	30/769	Mortality in hospital	Age, sex, hypertension, chronic renal disease, chronic lung disease, diabetes mellitus, cancer, cough, dyspnea, immunocompromised status, heart disease, diarrhea	Unclear	Logistic regression	C-statistic^b^: 0.79 Sensitivity^b^: 0% Specificity^b^: 100%	Low	Unclear	Low	High
Zhou et al	China	January 2nd until February 28th, 2020	68 / 763	ICU admission	Age, respiratory rate, systolic blood pressure, smoking status, fever, chronic kidney disease	Unclear	Nomogram	C-statistic (95% CI)^c^: 0.78 (0.68 to 0.87)	Low	Unclear	Low	High

^a^Performance measures from internal validation using cross-validation

^b^Performance measures from internal validation using random split of data set into development and testing datasets

^c^Numbers and performance measures from external validation in 44 patients (14 events)

### Validation of selected models in LMICs

The validation dataset included 11,338 individuals from 9 LMICs with a median (IQR) age of 43 (31–58) years, and 6611 were men. Most of these individuals were from Nigeria (3956, 34.9%), Zimbabwe (2185, 19.3%), Cameroon (1903, 16.8%), or Guinea (1848, 16.3%). In total, 514 patients were admitted to ICU, and 1063 in-hospital deaths were recorded (either at the ICU or any other department). A summary of the characteristics of the study population is presented in Table 2, and stratified characteristics of the study population for each country are presented in Supplemental Table 2. The percentages of missing data per variable ranged from less than 1% for sex to 52% for ICU admission (Supplemental Table 3). A comparison of baseline characteristics of our validation study data with characteristics of development datasets is shown in Supplemental Table 4. The median age in the studies by Berzuini et al. (71 years) and by Zhang et al. (61 years) was higher compared to our validation cohort restricted to LMICs (43 years). The percentage of mortality was also higher in the study by Berzuini et al. (27% compared to 10.9% in our LMIC data), but the percentage was lower in two other studies (6.4% for Wang et al. and 4.3% for Zhang et al.) and not reported by Zhou et al.

**Table 2:** Characteristic of the study population

Variables	Total population (11,338)
**Country (%)**	
Burkina Faso	352 (3.1)
Cameroon	1903 (16.8)
Democratic Republic of Congo	353 (3.1)
Guinea	1848 (16.3)
India	409 (3.6)
Niger	220 (1.9)
Nigeria	3956 (34.9)
Zambia	112 (1)
Zimbabwe	2185 (19.3)
**General characteristics**	
Age, years	43 (31, 58)
Men, (%)	6611 (58.8)
Smoking, (%)	79 (0.7)
**Comorbidities**	
Hypertension, (%)	2236 (19.7)
CHD, (%)	197 (1.7)
CKD, (%)	139 (1.2)
Pulmonary disease, (%)	68 (0.6)
Diabetes, (%)	1190 (10.5)
Cancer, (%)	39 (0.3)
Immunosuppression, (%)	293 (3.4)
Liver disease, (%)	42 (0.4)
**Symptoms/clinical presentation at admission**	
Respiratory rate, breaths/min	22 (19, 26)
Systolic blood pressure, mmHg	126 (114, 140)
Diastolic blood pressure, mmHg	80 (70, 90)
Oxygen saturation, %	96 (92, 98)
Heart rate, breaths/min	89 (80, 100)
Body temperature, °C	
≤ 37.2	6236 (55)
37.3–38.0	1059 (9.3)
> 38.0	697 (6.1)
Cough, (%)	4123 (36.4)
Dyspnea, (%)	2396 (21.1)
Diarrhea, (%)	426 (3.8)
**Outcomes**	
Mortality, (%)	1063 (9.4)
ICU admission, (%)	514 (4.5)

Data are median (interquartile range (IQR)) for continuous variables and number (percentage) for categorical variables from the original data. *CHD* coronary heart disease, *CKD* chronic kidney disease, *PaO2* partial pressure of oxygen, *ICU* intensive care unit

### Discrimination

**Fig. 2:**
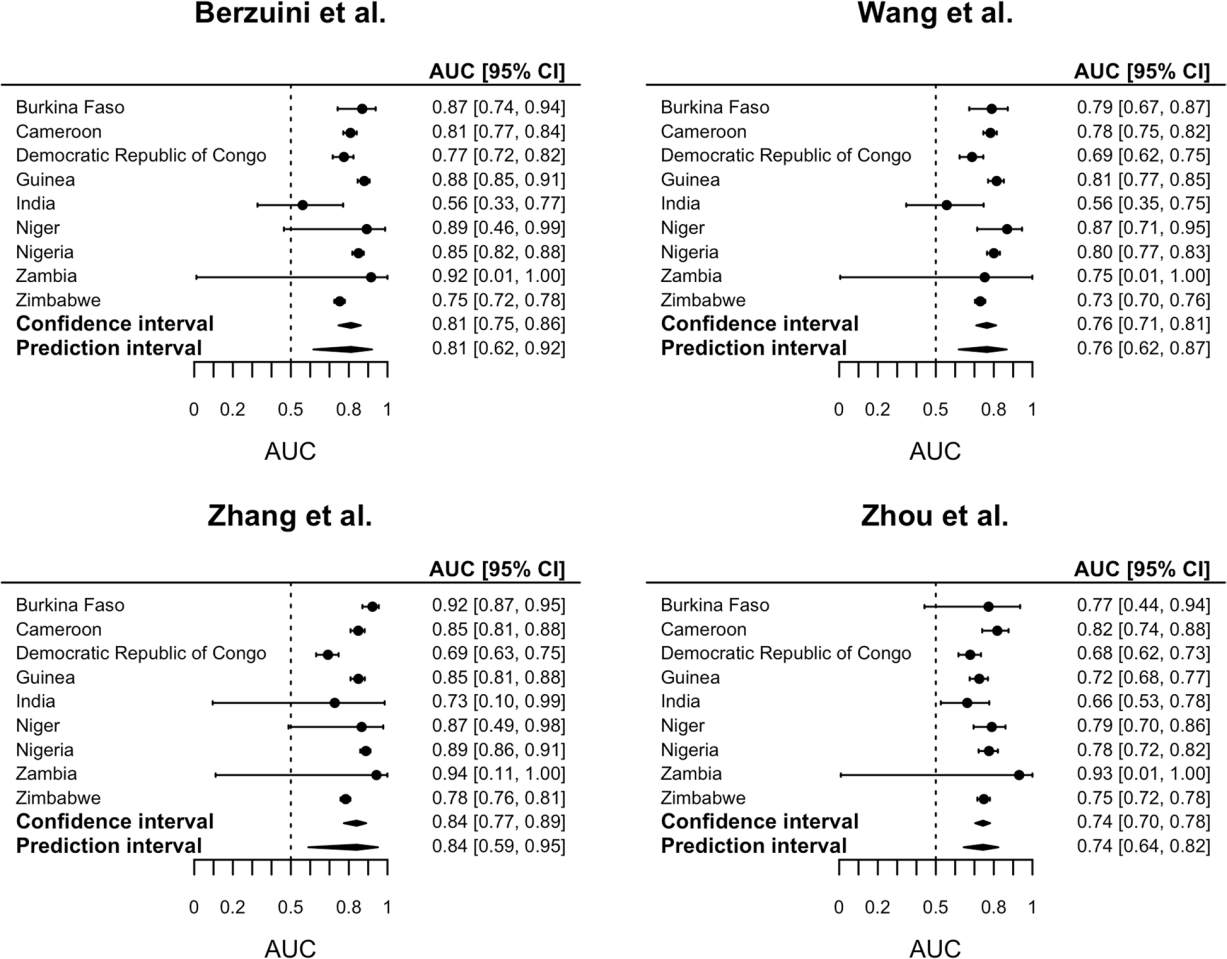
Discriminative performance of selected prediction models for predicting in-hospital mortality (Berzuini et al., Wang et al., Zhang et al.) or ICU admission (Zhou et al.)

Of the four included prediction models, the prediction model developed by Zhang et al. predicting the risk of in-hospital mortality was found to have the highest AUC of 0.84 (95% *CI* 0.77 to 0.89). The performance of this model was also the most heterogeneous, with a 95% PI for the AUC ranging between 0.59 and 0.95 (Fig. 2). This indicates a large fluctuation in performance of this model between LMICs. The discriminative performance of the other included prediction models for in-hospital mortality was slightly lower but again heterogeneous with an AUC of 0.81 (95% *CI* 0.75 to 0.86; 95% *PI* 0.62 to 0.92) for the model by Berzuini et al. and an AUC of 0.76 (95% *CI* 0.71 to 0.81; 95% *PI* 0.62 to 0.87) for the model by Wang et al. The model developed by Zhou et al. showed an AUC of 0.74 (95% *CI* 0.70 to 0.78) in predicting ICU admission risk, with large heterogeneity between studies (95% *PI* 0.64 to 0.82). A comparison of performance measures combined overimputed datasets using Rubin’s rules versus medians is presented in Supplemental Table 5.

### Observed to expected ratio and calibration-in-the-large

OE ratio was closest to the ideal value of 1 for the models by Wang et al. (1.32 [95% *CI* 0.82 to 2.12]) and Berzuini et al. (0.74 [95% *CI* 0.37 to 1.46]) (Supplemental Fig. 1). There was large variation between countries with 95% PI ranging from 0.35 to 4.99 for Wang et al. and 0.10 to 5.70 for Berzuini et al., indicating that in a new but similar validation, both underestimation or overestimation of risks can be expected. The Zhang et al. model underestimated the observed number of events in all countries with a pooled OE ratio of 4.36 (95% *CI* 3.08 to 6.17). The 95% PI for this model ranged between 1.71 and 11.10 indicating that underestimation is also expected in new but similar validation studies; however, there is a large variation in the extent of this underestimation. The model by Zhou et al. overestimated the risk of ICU admission with an OE ratio of 0.69, although the prediction interval was again wide and included both underestimation and overestimation (95% *CI* 0.46 to 1.04; 95% *PI* 0.20 to 2.35).

Similar results were found for calibration intercepts, which ranged between − 0.80 (95% *CI* − 1.56 to − 0.03; 95% *PI* − 3.09 to 1.50) for the model developed by Zhou et al. and 1.95 (95% *CI* 1.49 to 2.42; 95% *PI* 0.65 to 3.25) for the model by Zhang et al. (Supplemental Fig. 2).

### Calibration slope

For all four prognostic models, calibration slopes were below 1 (Supplemental Fig. 3), a characteristic that is consistent with overfitting of the prediction models. As a result, predictions made by the models are, on average, too extreme (i.e., low predicted risks are too low, and high predicted risks are too high). The calibration slope was closest to the ideal value of 1 for the model developed by Berzuini et al. (0.85 [95% *CI* 0.68 to 1.02]), but there was a large variation between countries (95% *PI* 0.45 to 1.25). A similar calibration slope was found for the model developed by Zhang et al. (0.79 [95% *CI* 0.64 to 0.95; 95% *PI* 0.37 to 1.22]). Results for the model by Zhou et al. were more consistent among countries, but this model showed considerable overfitting with a calibration slope of 0.37 (95% *CI* 0.31 to 0.43) and a prediction interval that does not include the ideal value of 1 (95% *PI* 0.24 to 0.51). This indicates that in new validation studies in LMICs, calibration slopes below 1 are expected. For the model by Wang et al., also a calibration slope below 1 was observed (0.55 [95% *CI* 0.46 to 0.64; 95% *PI* 0.32 to 0.78]).

**Fig. 3:**
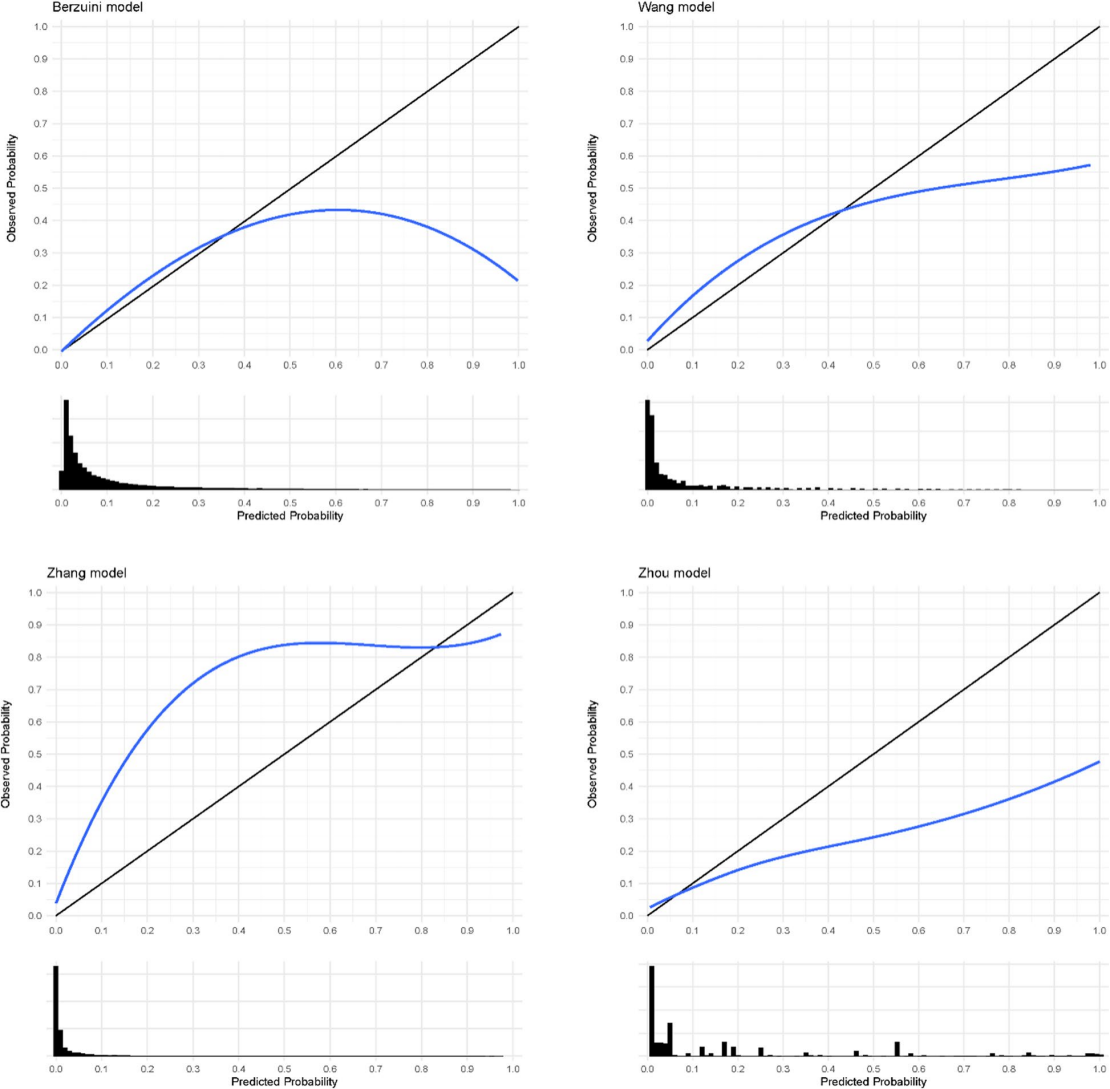
Calibration plots (observed vs predicted probabilities) of selected prediction models for predicting in-hospital mortality (Berzuini et al., Wang et al., Zhang et al.) or ICU admission (Zhou et al.). Calibration plots and loess lines were drawn in the stacked dataset (including all 50 imputed datasets) in the total population

### Calibration plots

The calibration plots show severe miscalibration for all four prognostic models (Fig. 3). For the models by Berzuini et al., Wang et al., and Zhou et al. we observed, especially for higher predicted risks, a substantial overestimation of risks (i.e., fewer events observed compared to what was predicted). Predicted risks by the model by Zhang et al. appeared to be underestimated over the full range of predicted risks. Calibration plots by country show considerable heterogeneity between countries (Supplemental Figs. 4, 5, 6, and 7).

## Discussion

Among 144 eligible models predicting ICU admission or mortality in hospitalized patients with suspected or confirmed COVID-19, only 4 did not have a high risk of bias in the PROBAST participant or prediction domain, used predictors available in our LMICs dataset and were reported in sufficient detail, and were therefore included for validation. In our individual participant data meta-analysis of 11,338 participants from 9 LMICs, we externally validated 3 models for the prediction of mortality and 1 model for ICU admission prediction. The selected models for the prediction of mortality showed, on average, a good ability to discriminate between hospitalized patients with confirmed COVID-19 for in-hospital mortality or ICU admission. However, there was extensive heterogeneity between countries. Good discrimination alone is not sufficient for a model to be used in practice, and calibration of the model should also be accurate [26]. The four prognostic models we validated all showed severe miscalibration with substantial heterogeneity between countries. If these models were used in clinical practice, this could lead to severe over- or underestimation of risks, potentially harmful to clinical decision-making. We advise that these models should not be used in LMICs without updating them.

### Comparison to previous literature

In an IPD-MA including 46,914 patients across 18 middle- and high-income countries [27], the Wang model obtained similar discriminative performance (AUC of 0.77 versus 0.76 in our study) and similar calibration slope (0.50 versus 0.55 in our study), while for the OE ratio, overestimation of risks was found (compared to, on average, no statistically significant uncerestimation in our study). The discriminative performance of the Zhang model was lower compared to our study (AUC of 0.70 versus 0.84), and in both IPD-MAs, predicted risks underestimated observed risks, which could potentially negatively affect clinical decision-making. Similar to our study, heterogeneity in performance between countries was found for all models. Validation studies of other models presented similar conclusions, e.g., a validation study of 22 prognostic models for patients admitted with COVID-19 concluded that none offered added value for patient risk stratification compared to using a single predictor [28]. It should also be noted that the performance of initially well-performing prognostic models can change over time due to the continuous changes in the COVID-19 virus, available treatments, and public measures to prevent infections [29].

With the higher burden of COVID-19 and limited medical resources in LMICs [6], use of accurate and reliable prognostic models could help guide efficient health policies and timely clinical decision-making [5, 9]. However, very few prognostic models have been developed in these settings [9]. In our study, the limited availability of predictors of developed prognostic models in our dataset of LMICs was an important factor in reducing the number of prognostic models we could validate. From 109 eligible prognostic models, 94 were excluded because not all predictors were present in our dataset or were missing for more than 80% of participants. This could be a reflection of reported limitations in laboratory testing and imaging availability in LMICs [9]. Thus, the applicability of available developed models and the feasibility of implementing them in LIMCs were limited. This highlights a real-world obstacle in the management of COVID-19: strategies executed in high-income countries may be unrealistic in most LMICs [30]. Further consideration on the availability, feasibility, and costs of predictors can help increase the generalizability of developed models and facilitate their extensive utilization across the world. Furthermore, the presentation of a basic version of developed models alongside the extended models seems helpful for use in LMICs.

### Strengths and limitations

We validated 4 prognostic models in a large dataset of more than 11,000 individuals from 9 LMICs. All study centers entered their data in a standardized WHO form for which explicit instructions were provided. The IPD design of our study allowed us to explore heterogeneity between countries. It, therefore, allowed a more complete impression of the transportability of these models compared to validation studies including data from a single center. A limitation is that all models were developed using data collected only during the first month of the COVID-19 pandemic, and the data in our IPD were collected early on in the pandemic. Since then, our knowledge of how to treat infected patients has increased. Furthermore, the COVID-19 virus has mutated, and its current characteristics are quite different from the beginning of the pandemic. As these prediction models have been developed and validated on data collected during the beginning of the pandemic, it is expected that performance on more recent data will be worse compared to our findings.

Another limitation is the high percentage of missing data for the outcome ICU admission. As there is a lack of metadata on the LMIC facilities that have contributed data, we cannot ascertain presence or absence of an ICU in these facilities.

We identified 15 prediction models that fulfilled our eligibility criteria, had a low risk of bias, and for which predictors were available in our dataset; however, unfortunately, we had to exclude 11 because we could not obtain the full regression equation necessary for external validation. In addition, the definition of immunosuppression used in the Zhang model was not reported, and we suspect differences between our definition and the definition used by Zhang et al. [24].

Furthermore, we validated models that are at high or unclear risk of bias for the domain participants and predictors, while actually one would prefer to focus on models at overall low risk of bias. Unfortunately, none of the 144 potentially eligible models was at overall low risk of bias, presented their full regression equation, ánd used predictors that are available in LMICs.

### Future directions

Future research should focus on developing new models using data collected from LMICs. In the systematic review, we used as a starting point for selection prediction models for validation, only one model was identified that was developed using data from an LMIC (Algeria) [31]. We could not validate this model as it included several blood-based tests as predictors that are not routinely collected in LIMCs, and, therefore also not available in our dataset. Out of the 144 eligible models, we could validate only four models, mainly because details for calculating individual risks were missing or predictors were not available in our dataset of LMICs. To use prognostic models for outcome prediction in LMICs, for COVID-19 or future pandemics, it is essential that models are tailored to low-resource settings. Furthermore, we advise to completely report details of development and validation studies, following the TRIPOD guidance [11, 12].

## Conclusion

Information about the prognosis of patients with COVID-19 is essential for individualized treatment decisions and resource use planning. We identified only four prediction models that could be applied to data collected from LMIC settings. Although the discriminative performance of these models was sufficient, the calibration of the risk predictions from these models was heterogeneous and generally very poor. These models should therefore not be used in clinical practice in LMICs.

## Supplementary Information

Additional file1: Supplemental Fig. 1. OE ratio of selected prediction models for predicting in-hospital mortalityor ICU admission. Supplemental Fig. 2. Calibration-in-the-large of selected prediction models for predicting in-hospital mortalityor ICU admission. Supplemental Fig. 3. Calibration slope of selected prediction models for predicting in-hospital mortalityor ICU admission. Supplemental Fig. 4. Calibration plots for predicted vs observed probabilities of mortality based on Berzuini et al. model by country. Supplemental Fig. 5. Calibration plots for predicted vs observed probabilities of mortality based on Wang et al.model by country. Supplemental Fig. 6. Calibration plots for predicted vs observed probabilities of mortality based on Zhang et al.model by country. Supplemental Fig. 7. Calibration plots for predicted vs observed probabilities of ICU admission based on Zhou et al. model by country. Supplemental Table 1. Risk of bias of eligible models. Supplemental Table 2. Characteristics of the study population by country. Supplemental Table 3. Numberof missing data by country. Supplemental Table 4. Comparison of baseline characteristics between development cohorts and validation cohort. Supplemental Table 5. Comparison of Rubin’s rules with medians for combining performance over imputed datasets

## Data Availability

Data collected for this study are not available for sharing.
