# Supplementary material for: Validation of prognostic models predicting mortality or ICU admission in patients with COVID-19 in low- and middle-income countries: a global individual participant data meta-analysis
Source: Diagn Progn Res. 2024 Dec 19;8:17. doi: 10.1186/s41512-024-00181-5 (PMC11656577; doi:10.1186/s41512-024-00181-5)
Supplement: Supplementary file 1 — Additional file1: Supplemental Fig. 1. OE ratio of selected prediction models for predicting in-hospital mortalityor ICU admission. Supplemental Fig. 2. Calibration-in-the-large of selected prediction models for predicting in-hospital mortalityor ICU admission. Supplemental Fig. 3. Calibration slope of selected prediction models for predicting in-hospital mortalityor ICU admission. Supplemental Fig. 4. Calibration plots for predicted vs observed probabilities of mortality based on Berzuini et al. model by country. Supplemental Fig. 5. Calibration plots for predicted vs observed probabilities of mortality based on Wang et al.model by country. Supplemental Fig. 6. Calibration plots for predicted vs observed probabilities of mortality based on Zhang et al.model by country. Supplemental Fig. 7. Calibration plots for predicted vs observed probabilities of ICU admission based on Zhou et al. model by country. Supplemental Table 1. Risk of bias of eligible models. Supplemental Table 2. Characteristics of the study population by country. Supplemental Table 3. Numberof missing data by country. Supplemental Table 4. Comparison of baseline characteristics between development cohorts and validation cohort. Supplemental Table 5. Comparison of Rubin’s rules with medians for combining performance over imputed datasets [file 41512_2024_181_MOESM1_ESM.docx]

**Supplemental File**

**Validation of prognostic models predicting mortality or ICU admission in patients with COVID-19 in low- and middle-income countries: a global individual participant data meta-analysis**

Johanna A.A. Damen, Banafsheh Arshi, Maarten van Smeden, Silvia Bertagnolio, Janet V. Diaz, Ronaldo Silva, Soe Soe Thwin, Laure Wynants, Karel G.M. Moons

Table of Contents

[Supplemental Methods: Characteristics of imputation models 3](#_Toc183505027)

[Supplemental Methods: Model equations 4](#_Toc183505028)

[Supplemental Methods: Definition of predictors and outcomes 5](#_Toc183505029)

[Supplemental Table 1. Risk of bias of eligible models 7](#_Toc183505030)

[Supplemental Table 2. Characteristics of the study population by country 11](#_Toc183505031)

[Supplemental Table 3. Number (%) of missing data by country 12](#_Toc183505032)

[Supplemental Table 4. Comparison of baseline characteristics between development cohorts and validation cohort 13](#_Toc183505033)

[Supplemental Table 5. Comparison of Rubin’s rules with medians for combining performance over imputed datasets 14](#_Toc183505034)

[Supplemental Figure 1. OE ratio of selected prediction models for predicting in-hospital mortality (Berzuini et al., Wang et al., Zhang et al.) or ICU admission (Zhou et al.). 16](#_Toc183505035)

[Supplemental Figure 2. Calibration-in-the-large of selected prediction models for predicting in-hospital mortality (Berzuini et al., Wang et al., Zhang et al.) or ICU admission (Zhou et al.) 17](#_Toc183505036)

[Supplemental Figure 3. Calibration slope of selected prediction models for predicting in-hospital mortality (Berzuini et al., Wang et al., Zhang et al.) or ICU admission (Zhou et al.) 18](#_Toc183505037)

[Supplemental Figure 4. Calibration plots for predicted vs observed probabilities of mortality based on Berzuini et al. model by country 19](#_Toc183505038)

[Supplemental Figure 5. Calibration plots for predicted vs observed probabilities of mortality based on Wang et al.model by country 20](#_Toc183505039)

[Supplemental Figure 6. Calibration plots for predicted vs observed probabilities of mortality based on Zhang et al.model by country 21](#_Toc183505040)

[Supplemental Figure 7. Calibration plots for predicted vs observed probabilities of ICU admission based on Zhou et al. model by country 22](#_Toc183505041)

# Supplemental Methods: Characteristics of imputation models

Data were imputed using joint modeling multiple imputation using the R package mitml. To account for the multilevel structure of the dataset, country was taken as the cluster variable. Other variable included in the imputation model were age, sex, smoking, hypertension, coronary heart disease, pulmonary disease, diabetes, cancer, liver disease, chronic kidney disease, immunosuppressive disorders, cough, dyspnea, temperature, oxygen saturation, respiratory rate, mortality and ICU admission. None of the included variables had completely observed data. After running 50,000 burn-in iterations, data were imputed 50 times with 5000 iterations between imputations. Convergence was checked visually.

# Supplemental Methods: Model equations

*Berzuini et al.(1)*

Logit(outcome) = -5.37 + 0.064 * age - 0.028 * O_2_ saturation + 0.085 * respiratory rate + 0.7 * smoking

The used equation is different from the published equation. After contacting the study authors because of abnormal predicted risks (mostly >50% predicted risk of mortality), it turned out that the wrong equation was reported and the correct equation was provided.

*Wang et al.(2)*

Logit(outcome) = -8.6 + 0.10 * age + 0.60 * hypertension + 1.11 * coronary heart disease

*Zhang et al.(3)*

Logit(outcome) = -8.5016 + 0.0461 * age + 0.3177 * male sex + 1.0975 * pulmonary disease +

0.4448 * diabetes + 1.3798 * immunocompromised + 0.0185 * cancer + 0.3457 * hypertension + 0.6761 * coronary heart disease + 1.3210 * chronic kidney disease + 0.7409 * cough + 1.5245 * dyspnea

*Zhou et al.(4)*

Logit(outcome) = -5.111 + 1.473 * temperature<37.2 + 0.984 8 temperature 37.3-39 + 2.170 * cough + 1.329 * dyspnea + 1.821 * hypertension + 3.232 * coronary heart disease + 2.232 * liver disease + 3.732 *chronic kidney disease

# Supplemental Methods: Definition of predictors and outcomes

| **Predictor** | **Definition / measurement details** |
| --- | --- |
| Age | Calculated from date of birth |
| Sex | Sex at birth |
| Smoking | Smoking at least one cigarette, cigar, pipe or equivalent per day before the onset of the current illness. Excluding smoke-free tobacco products such as chewed tobacco or electronic nicotine delivery devices. |
| Hypertension | One of the following:   - High blood pressure for which medication has been prescribed - Systolic blood pressure ≥140 mmHg - Diastolic blood pressure ≥90 mmHg |
| Coronary heart disease | Angina, ischemic heart disease, atherosclerotic heart disease, previous coronary artery bypass graft, previous cardiac stenting/coronary intervention |
| Pulmonary disease | Chronic obstructive pulmonary disease (also chronic obstructive airways disease, chronic bronchitis, emphysema), cystic fibrosis, bronchiectasis, interstitial lung disease (e.g. pulmonary fibrosis, asbestosis, autoimmune), or a pre-existing requirement for long-term oxygen therapy |
| Diabetes | Current diagnosis of or is being treated for type I or type II diabetes mellitus requiring oral or subcutaneous treatment |
| Cancer | Current solid organ or haematological malignancy. Excluding malignancies that have been declared “cured” ≥ 5 years ago with no evidence of ongoing disease, non-melanoma skin cancers, and benign growths or dysplasia. |
| Liver disease | Recorded at admission as yes/no/unclear. No further instructions provided. |
| Chronic kidney disease | Clinician-diagnosed chronic kidney disease, including any with:   - Markers of kidney damage (albuminuria, haematuria of renal origin, electrolyte abnormalities due to tubular disorders, renal histological abnormalities, structural abnormalities detected by imaging) - Estimated glomerular filtration rate < 60 mL/min/1.73 m2 - History of kidney transplantation. |
| Immunosuppressive disorders | One of the following:   - HIV: history of laboratory-confirmed HIV infection or AIDS-defining illness regardless of current viral load or CD4+ count. Regardless whether the patient is currently taking antiretroviral treatment. - Tuberculosis: receiving treatment for tuberculosis. Excluding latent tuberculosis, patients who have been cured of tuberculosis, and those who have chronic pulmonary sequelae following their tuberculosis. |
| Cough | Reported by the patient or observed on physical exam at admission |
| Dyspnea | Reported by the patient or observed on physical exam at admission |
| Temperature | First documented patient temperature, regardless of route (oral,  peripheral, etc.) in degrees Celsius |
| Oxygen saturation | First documented patient peripheral oxygen saturation measurement as a percentage, either while the patient was breathing room air or any form of supplemental oxygen. In case the pulse oximeter has given two different readings in succession, with no change to oxygen therapy, the greater measurement was recorded. |
| Respiratory rate | First documented patient respiratory rate in breaths/min |
| **Outcome** |  |
| Mortality | In-hospital mortality |
| ICU admission | Admitted to ICU or a high dependency unit at any point during their stay in hospital |

# Supplemental Table 1. Risk of bias of eligible models

|  | **Risk of bias domain** | | | | |
| --- | --- | --- | --- | --- | --- |
| **Reference** | **Participants** | **Predictors** | **Outcome** | **Analysis** | **Overall** |
| Abdulaal, Patel et al | Low | Low | Low | High | High |
| Abdulaal, Patel et al 2 | Low | Low | Low | High | High |
| Acar, Can et al | Low | Low | Low | High | High |
| Altschul, Unda et al | Low | Low | Low | High | High |
| Alvarez-Mon, Ortega et al | Unclear | Low | Unclear | High | High |
| Bellos, Lourida et al | Low | Low | Low | High | High |
| Bennouar, Cherif et al | High | Low | Low | High | High |
| Bertsimas, Lukin et al | Unclear | Low | Low | High | High |
| Berzuini, Hannan et al | Unclear | Low | Low | High | High |
| Berzuini, Hannan et al | Unclear | Low | Low | High | High |
| Berzuini, Hannan et al | Unclear | Low | Low | High | High |
| Binchen Wang, Feiyang Zhong | Low | Low | Low | High | High |
| Booth, Abels et al | Low | HIgh | Low | High | High |
| Bradley, Frost et al | Low | Unclear | Low | High | High |
| Bradley, Frost et al | Low | Unclear | Low | High | High |
| Cao, Li et al | Low | Unclear | Unclear | High | High |
| Cao, Zhang et al | Low | Unclear | Low | High | High |
| Carr, Bendayan et al | Low | Low | Low | High | High |
| Carr, Bendayan et al | low | Low | Low | Unclear | Unclear |
| Chen et al. Risk factors for mortality | Unclear | Unclear | Low | High | High |
| Chen et al. Risk factors for mortality | Unclear | Unclear | Low | High | High |
| Chen et al. Risk factors for mortality | Unclear | Low | Low | High | High |
| Chen, et al. Development and validation of a nomogram | Unclear | Low | Low | High | High |
| Chen, et al. Predicting severe or critical symptoms | High | Unclear | Low | High | High |
| Cheng, et al. Diagnostic performance of unitial blood urea nitrogen | Unclear | Unclear | Unclear | High | High |
| Cho et al. Prognosis score system | Low | Unclear | Low | High | High |
| Dai, Zeng et al | Low | Low | Unclear | High | High |
| Dai, Zeng et al | Low | Low | Unclear | High | High |
| Das, Mishra, et al | Low | Low | Low | High | High |
| Ding, Li et al | High | Low | Low | High | High |
| Doganci, Ince et al | High | Unclear | Low | High | High |
| Dong, Sun et al | Low | Unclear | Low | High | High |
| Ebrahimian, Homayounieh et al | High | Unclear | Low | High | High |
| Ebrahimian, Homayounieh et al | High | Unclear | Low | High | High |
| Fan, Hao et al | High | Low | Unclear | High | High |
| Fumagalli, Rozzini | Low | Low | Low | High | High |
| Galloway, Norton et al | Low | Low | Low | High | High |
| Gao, Cai et al | Low | Low | Low | Unclear | Unclear |
| Gidari, de Socio et al | Low | Low | Low | High | High |
| Gong, Ou et al. | Low | Unclear | Unclear | High | High |
| Guan, Zhang 2021 | Unclear | Unclear | Unclear | High | High |
| Gue, Tennyson 2020 | Low | Low | Low | High | High |
| Guo, Liu et al. | Low | High | Unclear | High | High |
| Guo, Xiong et al | Low | Low | High | High | High |
| Hachim, Hachim et al | Low | Low | High | High | High |
| Haimovich, Ravindra et al | Low | Low | High | High | High |
| Holten, Nore et al | low | Unclear | Low | high | High |
| Hong, Wu et al | Low | Unclear | Low | High | High |
| Hu, Du et al | High | unclear | High | High | High |
| Hu, Liu et al | High | Low | Low | Low | High |
| Hu, Liu et al | High | Low | Low | High | High |
| Hu, Ni et al | low | low | low | high | High |
| Huang, Cai, et al. | High | Unclear | High | High | High |
| Huang, Cheng et al | Low | Unclear | Low | High | High |
| Huang, Cheng et al | Low | Unclear | Low | High | High |
| Ikemura, Bellin et al | low | low | low | Unclear | Unclear |
| Jamal, Doi et al | Low | Low | Low | High | High |
| Ji, Zhang et al | Low | Low | Low | High | High |
| Jian, Kansal et al | low | low | low | high | High |
| Jimenez‑Solem, Petersen et al | Low | Unclear | Unclear | High | High |
| Junhong Wang, Hua Zhang | Low | Low | Low | High | High |
| Kaeuffer, Ruch, et al | Low | Unclear | Unclear | High | High |
| Kivrak, Guldogan, et al. | High | Unclear | Low | Unclear | Unclear |
| Ko, Chung, et al. | Unclear | Low | Low | High | High |
| Laguna-Goya, Utrero-Rico, et al | High | Low | Low | High | High |
| Levy, Richardson et al. | Low | Low | Low | High | High |
| Li, Zhang et al | Unclear | Low | Unclear | High | High |
| Li, Zhang, et al. | Unclear | Unclear | High | High | High |
| Liu et al, CD8+ T cels predicted the | High | Low | High | High | High |
| Liu et al, Evaluation of the risk pre | High | Low | Low | High | High |
| Liu et al: A nomogram for predi | High | Low | Low | High | High |
| Liu, Fang et al. | Low | Low | Low | High | High |
| Liu, Shi et al. | Low | Unclear | Unclear | High | High |
| Lui H et al. Development and valid | Low | Low | Low | High | High |
| Luo, Liu et al. | Unclear | Low | Low | High | High |
| Luo, Mao et al | low | Low | Unclear | High | High |
| Ma, Li et al | Unclear | Unclear | high | high | High |
| Ma, Wang et al | low | Low | Low | High | High |
| magro, Zuccaro et al | Low | low | low | Unclear | Unclear |
| Manocha, Kirzner et al | high | High | Low | high | high |
| McRae, Dapkins et al | Unclear | Unclear | High | High | High |
| McRae, Simmons et al. | High | High | High | High | High |
| Mei, Hu et al | Low | Low | Low | High | High |
| Mei, Hu et al | Low | Low | Low | Unclear | Unclear |
| Mei, Wang et al | Low | Unclear | Low | High | High |
| Nicholson, Wooster et al | Low | Low | Low | High | High |
| Niu, Zhan et al | Unclear | Low | Low | High | High |
| Pan, Cheng, et al | Unclear | Low | Low | High | High |
| Pan, Li, et al | Low | Low | Low | High | High |
| Parchure, Joshi, et al | Low | Low | Low | High | High |
| Qin, Liu, et al | High | Low | Unclear | High | High |
| Rechtman, Curtin, et al | Unclear | Low | Low | Unclear | Unclear |
| Ruoran Wang, Min He | Low | Low | Low | High | High |
| Sanchez-Montanes, Rodriquez-Belenguer et al. | Low | Low | Low | High | High |
| Satici, Dermikol et al | Low | Low | Low | High | High |
| Satici, Dermikol et al | Low | Low | Low | High | High |
| Song, Dong et al | Low | Low | Low | High | High |
| Soto-Mota, Marfil-Garza et al | High | Low | High | High | High |
| Sourij, Aziz et al | Low | Low | Low | High | High |
| Sun, Hong et al | High | Low | Low | High | High |
| Taiyao Wang, Aris Paschalidis | Low | High | Low | High | High |
| Taiyao Wang, Aris Paschalidis | Low | High | Low | High | High |
| Taiyao Wang, Aris Paschalidis | Low | Low | Low | High | High |
| Torres-Macho, Ryan et al | low | Low | low | high | High |
| Wang, Hou et al. | High | Low | Low | High | High |
| Wang, Zha et al. | Low | Low | Low | High | High |
| Wang, Zuo et al | Low | Low | Low | High | High |
| Wang, Zuo et al | low | low | Low | High | High |
| Wollenstein - Betech 2020 | Low | High | Low | Unclear | High |
| Wu, Du et al | unclear | low | low | High | High |
| Wu, Yang et al. 2020 | High | Low | High | High | High |
| Wu, Zhou et al. 2020 | High | Low | Low | High | High |
| Xie, Hungerford et al. | Low | Low | Low | High | High |
| Xue, Gan et al | Low | Low | Unclear | High | High |
| Yan, Zhang et al. | Low | High | Low | High | High |
| Yang, Shen, et al | Low | Low | Unclear | High | High |
| Yu, let, et al | Low | Low | Low | High | High |
| Yu, Zhu et al | Unclear | Low | Low | High | High |
| Yu, Zhu et al | Unclear | Low | Low | High | High |
| Yuan, Sun et al | Low | Low | Unclear | High | High |
| Zeng, Deng et al | Unclear | Low | Low | High | High |
| Zhang, Guo et al | High | Low | Low | High | High |
| Zhang, Guo et al | High | Low | Low | High | High |
| Zhang, Qin et al. | Low | Low | Unclear | High | High |
| Zhang, Shi et al | Low | Unclear | Low | High | High |
| Zhang, Shi et al | Low | Unclear | Low | High | High |
| Zhang, Shi et al | Low | Unclear | Low | High | High |
| Zhang, Wu et al | Low | Low | High | High | High |
| Zhang, Zhang et al | High | Unclear | Unclear | High | High |
| Zhang, Zhang et al | High | Unclear | Unclear | High | High |
| Zheng, Xu et al. | High | Low | Unclear | High | High |
| Zhihong Weng, Qiaosen Chen | Low | Low | Low | High | High |
| Zhou, He et al. | Low | Low | Low | High | High |
| Zhou, He et al. | Low | Unclear | Low | High | High |
| Zhou, Huang et al, | Low | Unclear | Low | High | High |
| Zhou, Qin et al. | Low | Unclear | Low | High | High |
| Zhou, Qin et al. | Low | Unclear | Unclear | High | High |
| Zhou, Qin et al. | Low | Unclear | Low | High | High |
| Zhou, Qin et al. | Low | Unclear | Unclear | High | High |
| Zhu, Ge, et al. | High | Low | Unclear | High | High |
| Zou, Li et al | High | Low | Low | High | High |

# Supplemental Table 2. Characteristics of the study population by country

|  | **Burkina Faso**  **(N=352)** | **Cameroon (N=1903)** | **Democratic Republic of Congo**  **(N=353)** | **Guinea**  **(N=1848)** | **India**  **(N=409)** | **Niger**  **(N=220)** | **Nigeria**  **(N=3956)** | **Zambia**  **(N=112)** | **Zimbabwe (N=2185)** |  |
| --- | --- | --- | --- | --- | --- | --- | --- | --- | --- | --- |
| Age, years | 42 (26, 56) | 45 (34, 58) | 58 (45, 67) | 42 (31, 58) | 26 (24, 30) | 42 (30, 58) | 40 (30, 54) | 40 (29, 50) | 51 (38, 64) |  |
| Men, (%) | 223 (63.4) | 1162 (61.3) | 242 (68.6) | 1207 (65.4) | 30 (7.4) | 129 (70.1) | 2402 (61.4) | 69 (62.2) | 1147 (52.6) |  |
| **Comorbidities** | | | | | | | | | | |
| Hypertension, (%) | 77 (21.9) | 251 (13.2) | 166 (47) | 367 (19.9) | 45 (11) | 45 (20.5) | 614 (15.5) | 26 (23.2) | 645 (29.5) |  |
| CHD, (%) | 5 (1.4) | 9 (0.5) | 32 (9.1) | 23 (1.2) | 3 (0.7) | 15 (6.8) | 28 (0.7) | 2 (1.8) | 80 (3.7) |  |
| CKD, (%) | 24 (6.8) | 1 (0.1) | 44 (12.5) | 3 (0.2) | 0 (0) | 5 (2.3) | 14 (0.4) | 0 (0) | 48 (2.2) |  |
| Pulmonary disease, (%) | 1 (0.3) | 5 (0.3) | 2 (0.6) | 18 (1) | 2 (0.5) | 6 (2.7) | 9 (0.2) | 2 (1.8) | 23 (1.1) |  |
| Diabetes, (%) | 28 (8) | 120 (6.3) | 105 (29.7) | 220 (11.9) | 8 (2) | 33 (15) | 298 (7.5) | 8 (7.1) | 370 (16.9) |  |
| Cancer, (%) | 0 (0) | 0 (0) | 7 (2) | 5 (0.3) | 3 (0.7) | 3 (1.4) | 8 (0.2) | 1 (0.9) | 12 (0.5) |  |
| Immunosuppression, (%) | 1 (0.5) | 17 (1.3) | 4 (1.8) | 19 (1.8) | 8 (2) | 4 (2.1) | 25 (0.8) | 22 (27.5) | 193 (9.9) |  |
| Liver disease | 10 (2.8) | 1 (0.1) | 8 (2.3) | 11 (0.6) | 1 (0.2) | 1 (0.5) | 8 (0.2) | 0 (0) | 2 (0.1) |  |
| Smoking, (%) | 0 (0) | 0 (0) | 9 (2.5) | 12 (0.6) | 0 (0) | 4 (1.8) | 23 (0.6) | 5 (4.5) | 26 (1.2) |  |
| **Symptoms/clinical presentation at admission** | | | | | | | | | | |
| Respiratory rate*, breaths/min | 20 (17, 23) | 26 (22, 34) | 28 (23, 34) | 22 (19, 25) | 16 (16, 18) | 76 (73, 78) | 22 (20, 24) | 20 (18, 20) | 23 (20, 30) |  |
| Systolic blood pressure, mmHg | 131 (120, 150) | 132.5 (118, 150) | 132 (120, 150) | 130 (120, 140) | 118 (110,124) | 130 (120, 140) | 123 (110, 140) | 131 (120, 148) | 127 (115, 142) |  |
| Diastolic blood pressure, mmHg | 83 (74, 91) | 83 (76, 93) | 84 (74, 94) | 80 (80,90) | 78 (72, 82) | 80 (70, 90) | 80 (70, 89) | 84 (77, 93) | 78 (70, 89) |  |
| Oxygen saturation, (%)* | 97 (95, 98) | 95 (90, 98) | 92 (81, 96) | 98 (95, 99) | 98 (98, 99) | 98 (95, 99) | 97 (92, 98) | 97 (96, 98) | 93 (86, 97) |  |
| Heart rate, beats/minute | 89 (78, 101) | 95 (82, 107) | 98 (87, 110) | 91 (82, 103) | 85 (82, 92) | 91 (78, 101) | 87 (78, 96) | 82 (74, 94) | 90 (80, 101) |  |
| Body temperature, °C s | | | | | | | | | | |
| ≤37.2 | 251 (71.3) | 817 (42.9) | 207 (58.6) | 883 (47.8) | 132 (32.3) | 149 (67.7) | 2607 (65.9) | 104 (92.9) | 1086 (49.7) |  |
| 37.3–38.0 | 61 (17.3) | 181 (9.5) | 53 (15) | 219 (11.9) | 76 (18.6) | 21 (9.5) | 321 (8.1) | 3 (2.7) | 124 (5.7) |  |
| >38.0 | 24 (6.8) | 165 (8.7) | 71 (20.1) | 147 (8) | 24 (5.9) | 11 (5) | 180 (4.6) | 3 (2.7) | 72 (3.3) |  |
| Cough, (%) | 106 (30.1) | 165 (8.7) | 217 (61.5) | 857 (46.4) | 48 (11.7) | 63 (28.6) | 1608 (40.6) | 19 (17) | 1040 (47.6) |  |
| Dyspnea, (%) | 72 (20.5) | 64 (3.4) | 302 (85.6) | 434 (23.5) | 17 (4.2) | 57 (25.9) | 465 (11.8) | 7 (6.2) | 978 (44.8) |  |
| Diarrhea, (%) | 3 (0.9) | 34 (1.8) | 22 (6.2) | 104 (5.6) | 6 (1.5) | 7 (3.2) | 102 (2.6) | 1 (0.9) | 147 (6.7) |  |
| **Outcomes** | | | | | | | | | | |
| Mortality, (%) | 21 (6) | 233 (12.2) | 110 (31.2) | 98 (5.3) | 7 (1.7) | 7 (3.2) | 186 (4.7) | 2 (1.8) | 399 (18.3) |  |
| ICU admission, (%) | 0 (0) | 0 (0) | 134 (38) | 81 (4.4) | 26 (6.4) | 30 (13.6) | 76 (1.9) | 2 (1.8) | 165 (7.6) |  |

CHD: coronary heart disease, CKD: chronic kidney disease, PaO2: Partial pressure of oxygen, ICU admission: intensive care unit admission. Data are mean (SD), * median (interquartile range (IQR)) for skewed variables, and number (percentage) for categorical variables from the original data

# Supplemental Table 3. Number (%) of missing data by country

|  | **Total population (N=11338)** | **Burkina Faso**  **(N=352)** | **Cameroon (N=1903)** | **Democratic Republic of Congo**  **(N=353)** | **Guinea**  **(N=1848)** | **India**  **(N=409)** | **Niger**  **(N=220)** | **Nigeria**  **(N=3956)** | **Zambia**  **(N=112)** | **Zimbabwe (N=2185)** |
| --- | --- | --- | --- | --- | --- | --- | --- | --- | --- | --- |
| Age, years | 1382 (12.2) | 4 (1.1) | 627 (33) | 4 (1.1) | 132 (7.1) | 67 (16.4) | 7 (3.2) | 420 (10.6) | 7 (6.3) | 114 (5.2) |
| Sex, (%) | 105 (0.9) | 0 (0) | 9 (0.5) | 0 (0) | 5 (0.3) | 5 (1.2) | 36 (16.4) | 43 (1.1) | 1 (0.9) | 6 (0.3) |
| Hypertension, (%) | 1645 (14.5) | 8 (2.3) | 661 (34.7) | 11 (3.1) | 37 (2) | 5 (1.2) | 40 (18.2) | 237 (6) | 1 (0.9) | 645 (29.5) |
| CHD, (%) | 2290 (20.2) | 8 (2.3) | 985 (51.8) | 11 (3.1) | 44 (2.4) | 4 (1) | 44 (20) | 368 (9.3) | 1 (0.9) | 825 (37.8) |
| CKD, (%) | 3201 (28.2) | 8 (2.3) | 1490 (78.3) | 10 (2.8) | 41 (2.2) | 4 (1) | 41 (18.6) | 772 (19.5) | 1 (0.9) | 834 (38.2) |
| Pulmonary disease, (%) | 2538 (22.4) | 8 (2.3) | 1016 (53.4) | 10 (2.8) | 42 (2.3) | 5 (1.2) | 44 (20) | 557 (14.1) | 1 (0.9) | 855 (39.1) |
| Diabetes, (%) | 1909 (16.8) | 10 (2.8) | 662 (34.8) | 7 (2) | 47 (2.5) | 1 (0.2) | 41 (18.6) | 382 (9.7) | 1 (0.9) | 758 (34.7) |
| Cancer, (%) | 3705 (32.7) | 8 (2.3) | 1626 (85.4) | 11 (3.1) | 60 (3.2) | 5 (1.2) | 56 (25.5) | 1053 (26.6) | 1 (0.9) | 885 (40.5) |
| Immunosuppression, (%) | 2344 (26.9) | 7 (3.4) | 677 (50.6) | 13 (5.9) | 39 (3.7) | 1 (0.3) | 37 (19.5) | 752 (22.9) | 1 (1.2) | 817 (41.9) |
| Liver disease, (%) | 3433 (30.3) | 9 (2.6) | 1492 (78.4) | 12 (3.4) | 38 (2.1) | 0 (0) | 42 (19.1) | 975 (24.6) | 1 (0.9) | 864 (39.5) |
| Smoking, (%) | 3348 (29.5) | 8 (2.3) | 1585 (83.3) | 14 (4) | 39 (2.1) | 3 (0.7) | 81 (36.8) | 727 (18.4) | 2 (1.8) | 889 (40.7) |
| Respiratory rate, breaths/min | 6406 (56.5) | 334 (94.9) | 1740 (91.4) | 32 (9.1) | 855 (46.3) | 14 (3.4) | 217 (98.6) | 1986 (50.2) | 7 (6.2) | 1221 (55.9) |
| Systolic blood pressure, mmHg | 4656 (41.1) | 28 (8) | 1467 (77.1) | 23 (6.5) | 701 (37.9) | 32 (7.8) | 62 (28.2) | 1388 (35.1) | 5 (4.5) | 950 (43.5) |
| Diastolic blood pressure, mmHg | 4728 (41.7) | 29 (8.2) | 1503 (79) | 24 (6.8) | 699 (37.8) | 30 (7.3) | 70 (31.8) | 1410 (35.6) | 5 (4.5) | 958 (43.8) |
| Oxygen saturation, (%) | 5099 (45.0) | 26 (7.4) | 1337 (70.3) | 52 (14.7) | 731 (39.6) | 5 (1.2) | 69 (31.4) | 1881 (47.5) | 6 (5.4) | 992 (45.4) |
| Heart rate, beats/minute | 5428 (47.9) | 38 (10.8) | 1421 (74.7) | 17 (4.8) | 703 (38) | 9 (2.2) | 94 (42.7) | 2130 (53.8) | 4 (3.6) | 1012 (46.3) |
| Body temperature, °C | 3346 (29.5) | 16 (4.5) | 740 (38.9) | 22 (6.2) | 599 (32.4) | 177 (43.3) | 39 (17.7) | 848 (21.4) | 2 (1.8) | 903 (41.3) |
| Cough, (%) | 1612 (14.2) | 1 (0.3) | 1001 (52.6) | 0 (0) | 0 (0) | 0 (0) | 2 (0.9) | 59 (1.5) | 0 (0) | 549 (25.1) |
| Dyspnea, (%) | 1680 (14.8) | 1 (0.3) | 1033 (54.3) | 0 (0) | 2 (0.1) | 4 (1) | 6 (2.7) | 144 (3.6) | 0 (0) | 490 (22.4) |
| Diarrhea, (%) | 2967 (26.2) | 4 (1.1) | 1112 (58.4) | 2 (0.6) | 16 (0.9) | 0 (0) | 40 (18.2) | 933 (23.6) | 2 (1.8) | 858 (39.3) |
| Mortality, (%) | 1552 (13.7) | 6 (1.7) | 12 (0.6) | 48 (13.6) | 37 (2) | 4 (1) | 20 (9.1) | 788 (19.9) | 5 (4.5) | 632 (28.9) |
| ICU admission, (%) | 5902 (52.1) | 349 (99.1) | 1719 (90.3) | 48 (13.6) | 1289 (69.8) | 10 (2.4) | 17 (7.7) | 1198 (30.3) | 4 (3.6) | 1268 (58) |

CHD: coronary heart disease, CKD: chronic kidney disease, ICU admission: intensive care unit admission

# Supplemental Table 4. Comparison of baseline characteristics between development cohorts and validation cohort

|  | **Validation (n=11338)** | **Berzuini et al. (n=392)** | **Wang et al. (n=296)** | **Zhang et al. (n=775)** | **Zhou et al. (n=366)** |
| --- | --- | --- | --- | --- | --- |
| **General characteristics** | | | | | |
| Age, years | 43 (31, 58) | 71 (22) | Mean (SD): 47.32 (14.95) | 61 (50-68) | 43 (31.8-51) |
| Men, (%) | 6611 (58.9) | 255 (65) | 140 (47.3) | 379 (48.9) | 207 (56.6) |
| Smoking, (%) | 79 (1) | 24 (7) | 12 (4) |  |  |
| **Comorbidities** |  |  |  |  |  |
| Hypertension, (%) | 2236 (23.1) |  | 42 (14.2) | 239 (30.8) | 38 (10.4) |
| CHD, (%) | 197 (2.2) |  | 10 (3.4) | 85 (11) |  |
| CKD, (%) | 139 (1.7) | 45 (12) |  | 25 (3.2) | 4 (1.1) |
| Pulmonary disease, (%) | 68 (0.8) |  |  | 48 (6.2) |  |
| Diabetes, (%) | 1190 (12.6) | 95 (24) | 30 (10.1) | 106 (13.7) | 21 (5.7) |
| Cancer, (%) | 39 (0.5) |  | 1 (0.3) | 24 (3.1) | 1 (0.3) |
| Liver disease, (%) |  | 14 (2) |  |  | 8 (2.2) |
| Immunosuppression, (%) | 293 (4.6) |  |  | 12 (1.5) |  |
| **Symptoms/clinical presentation at admission** | | | | | |
| Respiratory rate, breaths/min | 22.0 (19, 26) | Rate >24: 109 (30%) |  |  | Rate ≥25: 74 (20.2) |
| Systolic blood pressure, mmHg | 126 (114, 140) | MAP <70: 30 (8%) |  |  | ≥110: 337 (92.1) |
| Diastolic blood pressure, mmHg | 80 (70, 90) |  |  |  |  |
| Oxygen saturation, % | 96 (92, 98) | Saturation <90%: 59 (17%) | 97.0 (95.0, 99) |  | Saturation <96%: 64 (17.5%) |
| Heart rate, breaths/minute | 89 (80, 100) |  |  |  |  |
| Body temperature, °C |  |  |  |  |  |
| ≤37.2 | 6236 (78) |  |  |  | 258 (70.5) |
| 37.3–38.0 | 1059 (13.3) |  |  |  | 37 (10.1) |
| >38.0 | 697 (8.7) | 30 (8) | Fever: 213 (73.5%) | Fever: 532 (68.6%) | 71 (19.4) |
| Cough, (%) | 4123 (42.4) | 240 (61) | 197 (67) | 528 (68.1) | 115 (31.4) |
| Dyspnea, (%) | 2396 (24.8) | 245 (65) |  | 355 (45.8) | 23 (6.3) |
| Diarrhea, (%) | 426 (5.1) |  |  | 36 (4.6) |  |
| **Outcomes** |  |  |  |  |  |
| Mortality, (%) | 1063 (10.9) | 110 (27) | 19 (6.4) | 33 (4.3) |  |
| ICU admission, (%) | 514 (9.5) | 31 (12) |  |  |  |

# Supplemental Table 5. Comparison of Rubin’s rules with medians for combining performance over imputed datasets

|  | **Berzuini** | | **Wang** | | **Zhang** | | **Zhou** | |
| --- | --- | --- | --- | --- | --- | --- | --- | --- |
| **Country** | **Rubin’s (95% CI)** | **Median (IQR)** | **Rubin’s (95% CI)** | **Median (IQR)** | **Rubin’s (95% CI)** | **Median (IQR)** | **Rubin’s (95% CI)** | **Median (IQR)** |
| **AUC** | | | | | | | | |
| Burkina Faso | 0.87 (0.74 to 0.94) | 0.87 (0.86 to 0.88) | 0.79 (0.67 to 0.87) | 0.79 (0.79 to 0.79) | 0.92 (0.87 to 0.95) | 0.92 (0.92 to 0.93) | 0.77 (0.44 to 0.94) | 0.77 (0.72 to 0.81) |
| Cameroon | 0.81 (0.77 to 0.84) | 0.81 (0.80 to 0.81) | 0.78 (0.75 to 0.82) | 0.78 (0.78 to 0.79) | 0.85 (0.81 to 0.88) | 0.85 (0.84 to 0.86) | 0.82 (0.74 to 0.88) | 0.82 (0.80 to 0.83) |
| Democratic Republic of Congo | 0.77 (0.72 to 0.82) | 0.78 (0.77 to 0.78) | 0.69 (0.62 to 0.75) | 0.69 (0.68 to 0.69) | 0.69 (0.63 to 0.75) | 0.69 (0.69 to 0.70) | 0.68 (0.62 to 0.73) | 0.68 (0.67 to 0.68) |
| Guinea | 0.88 (0.85 to 0.91) | 0.88 (0.88 to 0.88) | 0.81 (0.77 to 0.85) | 0.82 (0.81 to 0.82) | 0.85 (0.81 to 0.88) | 0.85 (0.85 to 0.85) | 0.72 (0.68 to 0.77) | 0.73 (0.71 to 0.74) |
| India | 0.56 (0.33 to 0.77) | 0.55 (0.52 to 0.61) | 0.56 (0.35 to 0.75) | 0.55 (0.54 to 0.56) | 0.73 (0.10 to 0.99) | 0.69 (0.68 to 0.79) | 0.66 (0.53 to 0.78) | 0.66 (0.64 to 0.68) |
| Niger | 0.89 (0.46 to 0.99) | 0.89 (0.86 to 0.91) | 0.87 (0.71 to 0.95) | 0.87 (0.85 to 0.89) | 0.87 (0.49 to 0.98) | 0.86 (0.84 to 0.89) | 0.79 (0.70 to 0.86) | 0.79 (0.77 to 0.80) |
| Nigeria | 0.85 (0.82 to 0.88) | 0.85 (0.85 to 0.85) | 0.80 (0.77 to 0.83) | 0.80 (0.80 to 0.81) | 0.89 (0.86 to 0.91) | 0.89 (0.88 to 0.89) | 0.78 (0.72 to 0.82) | 0.78 (0.77 to 0.78) |
| Zambia | 0.92 (0.01 to 1.00) | 0.92 (0.85 to 0.96) | 0.75 (0.01 to 1.00) | 0.76 (0.69 to 0.83) | 0.94 (0.11 to 1.00) | 0.95 (0.93 to 0.96) | 0.93 (0.01 to 1.00) | 0.93 (0.93 to 0.93) |
| Zimbabwe | 0.75 (0.72 to 0.78) | 0.75 (0.75 to 0.76) | 0.73 (0.70 to 0.76) | 0.73 (0.73 to 0.73) | 0.78 (0.76 to 0.81) | 0.78 (0.78 to 0.79) | 0.75 (0.72 to 0.78) | 0.75 (0.74 to 0.75) |
| **CITL** | | | | | | | | |
| Burkina Faso | -0.05 (-0.62 to 0.53) | -0.05 (-0.16 to 0.06) | 0.14 (-0.35 to 0.63) | 0.13 (0.12 to 0.14) | 1.82 (1.32 to 2.31) | 1.82 (1.80 to 1.84) | -1.74 (-4.16 to 0.69) | -1.79 (-2.52 to -0.99) |
| Cameroon | -0.20 (-0.39 to -0.00) | -0.20 (-0.24 to -0.17) | 0.78 (0.60 to 0.96) | 0.78 (0.76 to 0.80) | 2.79 (2.58 to 3.00) | 2.79 (2.75 to 2.84) | -2.37 (-3.88 to -0.86) | -2.28 (-2.93 to -1.84) |
| Democratic Republic of Congo | 0.77 (0.50 to 1.04) | 0.78 (0.75 to 0.79) | 1.49 (1.22 to 1.76) | 1.50 (1.46 to 1.51) | 2.48 (2.22 to 2.75) | 2.48 (2.47 to 2.51) | -0.13 (-0.44 to 0.19) | -0.13 (-0.17 to -0.11) |
| Guinea | -0.44 (-0.67 to -0.22) | -0.44 (-0.46 to -0.43) | -0.09 (-0.32 to 0.14) | -0.09 (-0.10 to -0.08) | 1.66 (1.43 to 1.89) | 1.66 (1.65 to 1.67) | 0.02 (-0.29 to 0.33) | 0.01 (-0.08 to 0.12) |
| India | 0.04 (-0.79 to 0.87) | 0.05 (-0.02 to 0.10) | 1.36 (0.58 to 2.14) | 1.37 (1.33 to 1.40) | 2.35 (1.59 to 3.12) | 2.36 (2.33 to 2.38) | 0.49 (0.04 to 0.94) | 0.48 (0.44 to 0.53) |
| Niger | -4.40 (-5.69 to -3.12) | -4.43 (-4.67 to -4.08) | -1.09 (-1.92 to -0.26) | -1.08 (-1.20 to -0.99) | 0.71 (-0.16 to 1.59) | 0.72 (0.57 to 0.86) | -0.22 (-0.82 to 0.37) | -0.23 (-0.34 to -0.11) |
| Nigeria | -0.39 (-0.55 to -0.22) | -0.39 (-0.40 to -0.37) | 0.01 (-0.16 to 0.18) | 0.01 (-0.01 to 0.03) | 1.89 (1.71 to 2.06) | 1.88 (1.85 to 1.91) | -1.94 (-2.26 to -1.63) | -1.91 (-1.99 to -1.86) |
| Zambia | -0.79 (-2.22 to 0.64) | -0.80 (-0.85 to -0.78) | -0.80 (-2.24 to 0.65) | -0.82 (-0.84 to -0.81) | 1.05 (-0.39 to 2.48) | 1.02 (0.97 to 1.07) | -1.49 (-3.15 to 0.16) | -1.54 (-1.58 to -1.54) |
| Zimbabwe | 0.71 (0.58 to 0.84) | 0.71 (0.69 to 0.73) | 1.14 (1.00 to 1.28) | 1.14 (1.11 to 1.16) | 1.86 (1.70 to 2.02) | 1.86 (1.83 to 1.89) | -1.23 (-1.54 to -0.92) | -1.21 (-1.29 to -1.14) |
| **Calibration slope** | | | | | | | | |
| Burkina Faso | 1.18 (0.68 to 1.68) | 1.18 (1.08 to 1.27) | 0.64 (0.35 to 0.93) | 0.64 (0.63 to 0.65) | 1.06 (0.66 to 1.46) | 1.07 (1.00 to 1.11) | 0.37 (0.11 to 0.64) | 0.37 (0.32 to 0.43) |
| Cameroon | 0.82 (0.68 to 0.95) | 0.81 (0.79 to 0.84) | 0.58 (0.48 to 0.68) | 0.58 (0.57 to 0.60) | 0.84 (0.69 to 0.99) | 0.84 (0.80 to 0.87) | 0.39 (0.28 to 0.49) | 0.39 (0.37 to 0.40) |
| Democratic Republic of Congo | 0.73 (0.53 to 0.94) | 0.74 (0.72 to 0.76) | 0.39 (0.24 to 0.55) | 0.39 (0.38 to 0.40) | 0.52 (0.33 to 0.72) | 0.53 (0.50 to 0.54) | 0.26 (0.16 to 0.36) | 0.26 (0.25 to 0.27) |
| Guinea | 1.05 (0.87 to 1.24) | 1.05 (1.03 to 1.07) | 0.67 (0.54 to 0.80) | 0.67 (0.66 to 0.68) | 0.87 (0.71 to 1.02) | 0.86 (0.85 to 0.88) | 0.44 (0.34 to 0.55) | 0.44 (0.42 to 0.47) |
| India | 0.17 (-0.60 to 0.93) | 0.17 (0.13 to 0.22) | 0.17 (-0.74 to 1.09) | 0.17 (0.12 to 0.21) | 1.02 (0.24 to 1.80) | 1.01 (0.93 to 1.09) | 0.44 (0.16 to 0.71) | 0.44 (0.39 to 0.46) |
| Niger | 1.18 (0.37 to 1.99) | 1.17 (1.03 to 1.23) | 0.74 (0.23 to 1.24) | 0.73 (0.64 to 0.81) | 0.77 (0.25 to 1.28) | 0.76 (0.67 to 0.88) | 0.34 (0.17 to 0.51) | 0.34 (0.31 to 0.37) |
| Nigeria | 0.95 (0.83 to 1.07) | 0.95 (0.93 to 0.97) | 0.62 (0.54 to 0.71) | 0.62 (0.61 to 0.63) | 0.95 (0.84 to 1.06) | 0.95 (0.94 to 0.96) | 0.44 (0.36 to 0.52) | 0.44 (0.42 to 0.46) |
| Zambia | 1.73 (-0.83 to 4.30) | 1.59 (1.09 to 2.33) | 0.47 (-0.64 to 1.58) | 0.45 (0.31 to 0.66) | 1.62 (-0.25 to 3.50) | 1.60 (1.29 to 1.97) | 0.82 (0.14 to 1.50) | 0.81 (0.79 to 0.81) |
| Zimbabwe | 0.65 (0.55 to 0.74) | 0.64 (0.63 to 0.67) | 0.45 (0.38 to 0.51) | 0.44 (0.44 to 0.46) | 0.57 (0.49 to 0.65) | 0.57 (0.56 to 0.58) | 0.33 (0.28 to 0.39) | 0.34 (0.32 to 0.34) |
| **OE ratio** | | | | | | | | |
| Burkina Faso | 0.96 (0.59 to 1.57) | 0.96 (0.88 to 1.05) | 1.11 (0.73 to 1.69) | 1.11 (1.10 to 1.12) | 4.43 (2.89 to 6.79) | 4.49 (4.26 to 4.61) | 0.40 (0.10 to 1.54) | 0.39 (0.26 to 0.61) |
| Cameroon | 0.88 (0.76 to 1.01) | 0.87 (0.85 to 0.89) | 1.67 (1.46 to 1.90) | 1.67 (1.64 to 1.69) | 6.98 (5.63 to 8.64) | 6.93 (6.62 to 7.46) | 0.37 (0.19 to 0.71) | 0.38 (0.29 to 0.47) |
| Democratic Republic of Congo | 1.50 (1.29 to 1.75) | 1.51 (1.49 to 1.52) | 2.38 (2.04 to 2.76) | 2.38 (2.34 to 2.40) | 5.48 (4.60 to 6.53) | 5.54 (5.36 to 5.66) | 0.96 (0.84 to 1.10) | 0.96 (0.95 to 0.97) |
| Guinea | 0.71 (0.58 to 0.86) | 0.71 (0.70 to 0.72) | 0.93 (0.77 to 1.13) | 0.93 (0.92 to 0.94) | 4.02 (3.29 to 4.92) | 4.03 (3.98 to 4.10) | 1.01 (0.84 to 1.22) | 1.00 (0.95 to 1.07) |
| India | 1.04 (0.49 to 2.22) | 1.04 (0.98 to 1.09) | 3.69 (1.74 to 7.83) | 3.71 (3.57 to 3.87) | 9.95 (4.73 to 20.94) | 10.04 (9.68 to 10.33) | 1.45 (1.00 to 2.11) | 1.44 (1.40 to 1.49) |
| Niger | 0.07 (0.03 to 0.16) | 0.07 (0.06 to 0.08) | 0.45 (0.22 to 0.92) | 0.45 (0.42 to 0.49) | 1.77 (0.83 to 3.75) | 1.79 (1.59 to 1.99) | 0.91 (0.65 to 1.27) | 0.91 (0.86 to 0.95) |
| Nigeria | 0.74 (0.65 to 0.86) | 0.74 (0.73 to 0.76) | 1.01 (0.88 to 1.16) | 1.01 (0.99 to 1.02) | 4.24 (3.63 to 4.95) | 4.23 (4.10 to 4.39) | 0.34 (0.28 to 0.41) | 0.34 (0.33 to 0.35) |
| Zambia | 0.48 (0.12 to 1.91) | 0.47 (0.45 to 0.48) | 0.49 (0.12 to 1.94) | 0.48 (0.47 to 0.49) | 2.73 (0.69 to 10.84) | 2.67 (2.55 to 2.79) | 0.37 (0.09 to 1.48) | 0.36 (0.35 to 0.36) |
| Zimbabwe | 1.53 (1.40 to 1.66) | 1.53 (1.51 to 1.55) | 1.86 (1.71 to 2.03) | 1.87 (1.84 to 1.88) | 2.81 (2.53 to 3.13) | 2.80 (2.74 to 2.87) | 0.64 (0.57 to 0.73) | 0.64 (0.63 to 0.66) |

# Supplemental Figure 1. OE ratio of selected prediction models for predicting in-hospital mortality (Berzuini et al., Wang et al., Zhang et al.) or ICU admission (Zhou et al.).


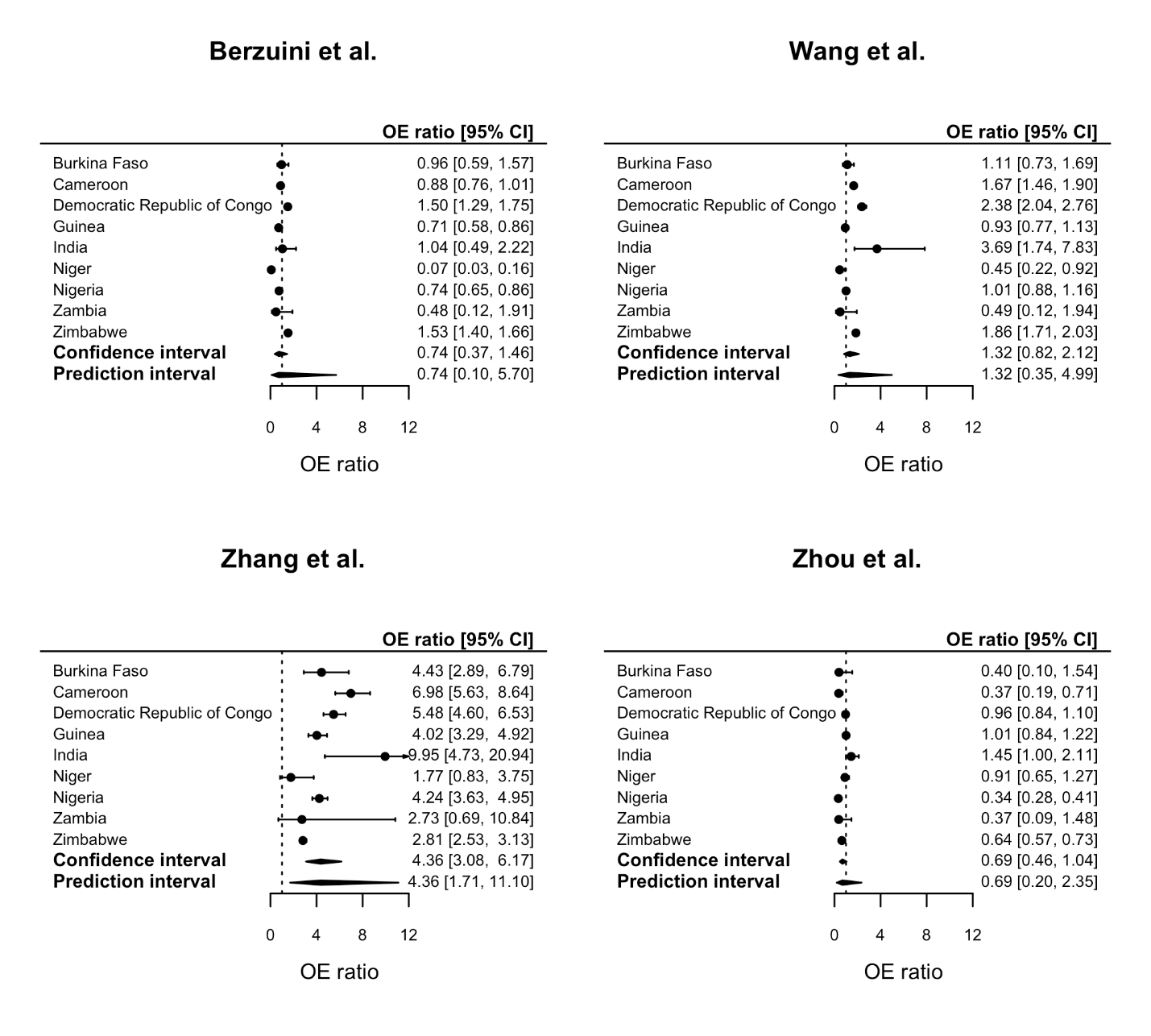


# Supplemental Figure 2. Calibration-in-the-large of selected prediction models for predicting in-hospital mortality (Berzuini et al., Wang et al., Zhang et al.) or ICU admission (Zhou et al.)


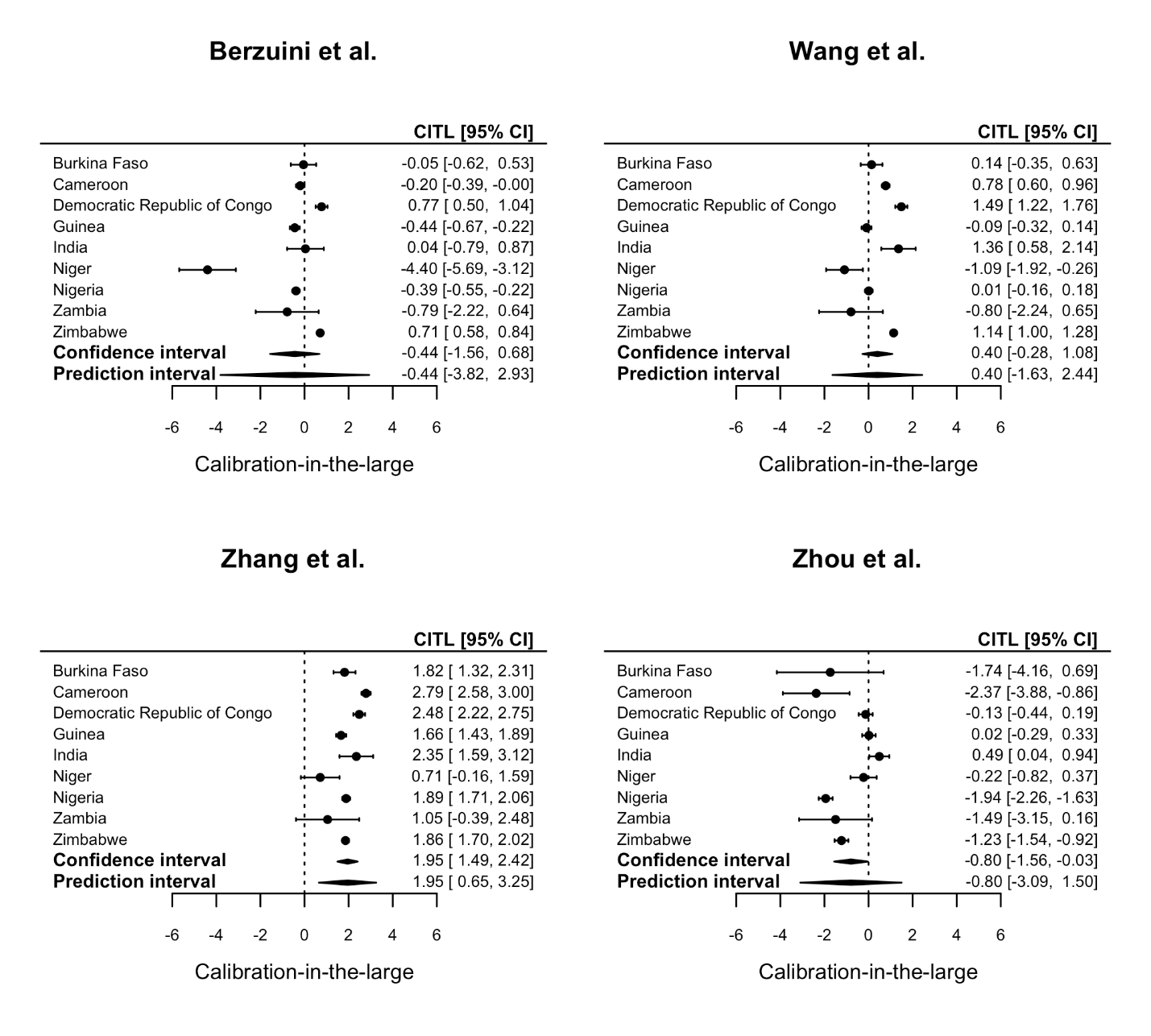


# Supplemental Figure 3. Calibration slope of selected prediction models for predicting in-hospital mortality (Berzuini et al., Wang et al., Zhang et al.) or ICU admission (Zhou et al.)


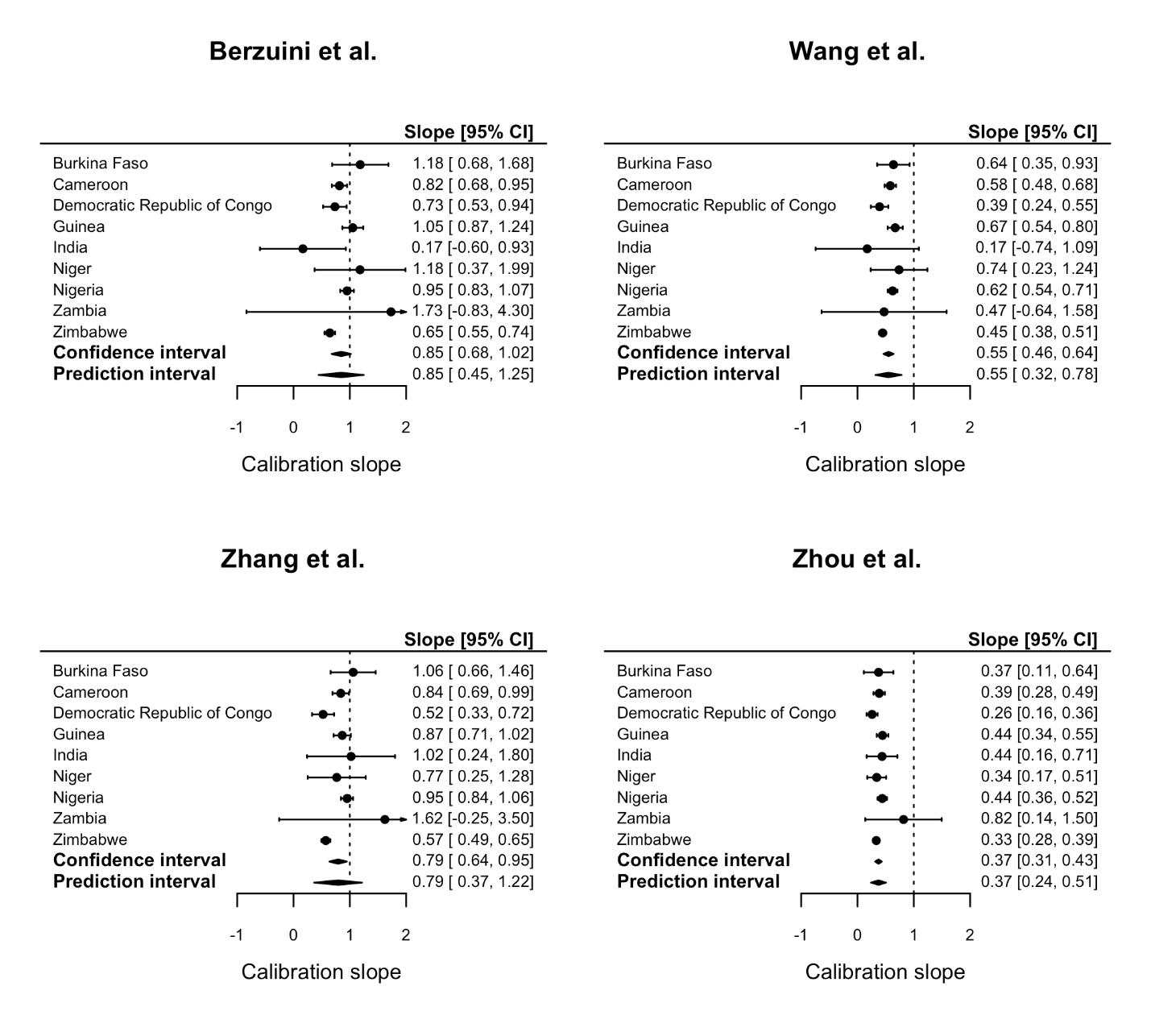


# Supplemental Figure 4. Calibration plots for predicted vs observed probabilities of mortality based on Berzuini et al. model by country


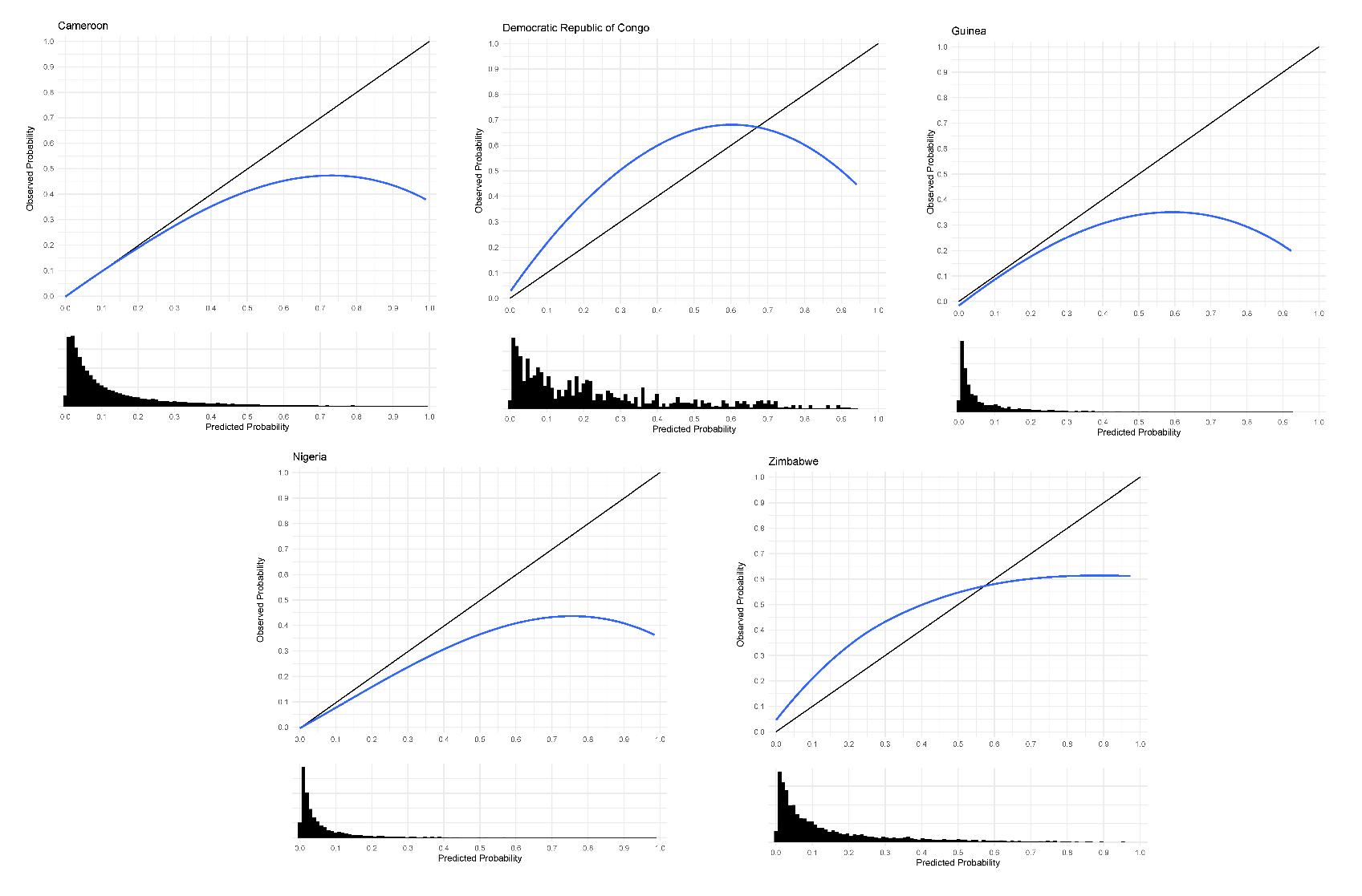


Calibration plots and loess lines were drawn in the stacked dataset (including all 50 imputed datasets) in the total population.

# Supplemental Figure 5. Calibration plots for predicted vs observed probabilities of mortality based on Wang et al.model by country


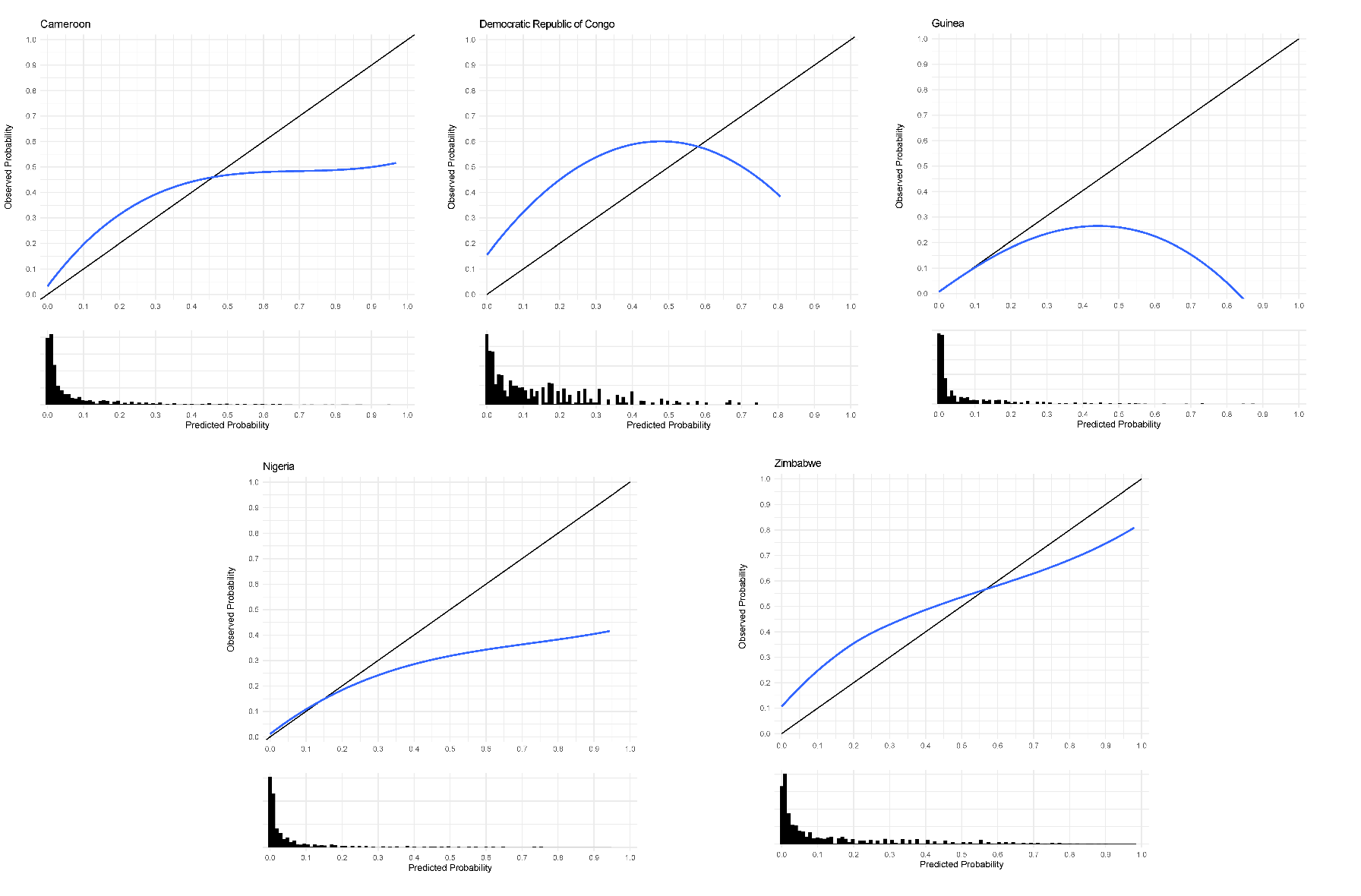


Calibration plots and loess lines were drawn in the stacked dataset (including all 50 imputed datasets) in the total population.

# Supplemental Figure 6. Calibration plots for predicted vs observed probabilities of mortality based on Zhang et al.model by country


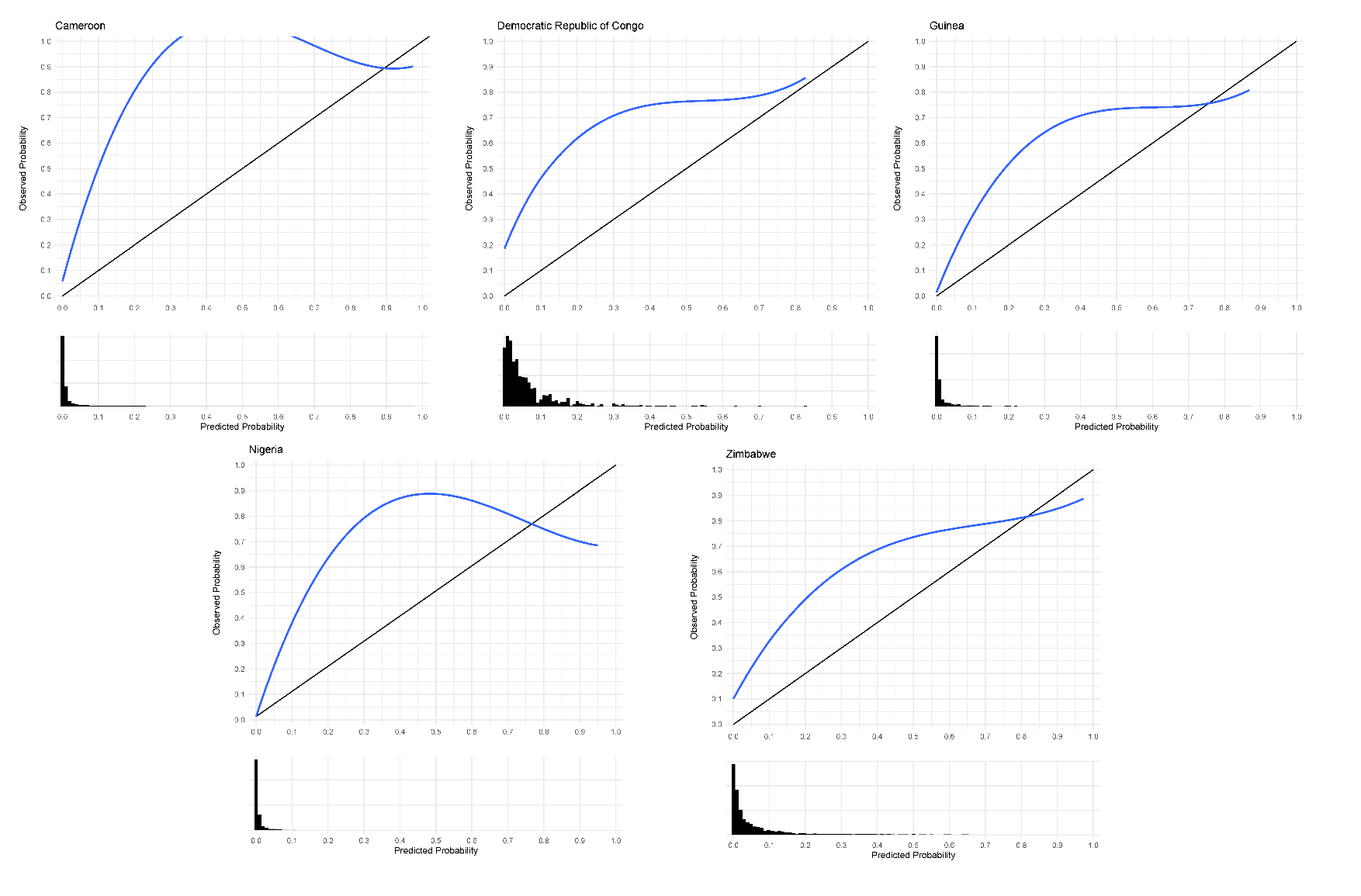


Calibration plots and loess lines were drawn in the stacked dataset (including all 50 imputed datasets) in the total population.

# Supplemental Figure 7. Calibration plots for predicted vs observed probabilities of ICU admission based on Zhou et al. model by country


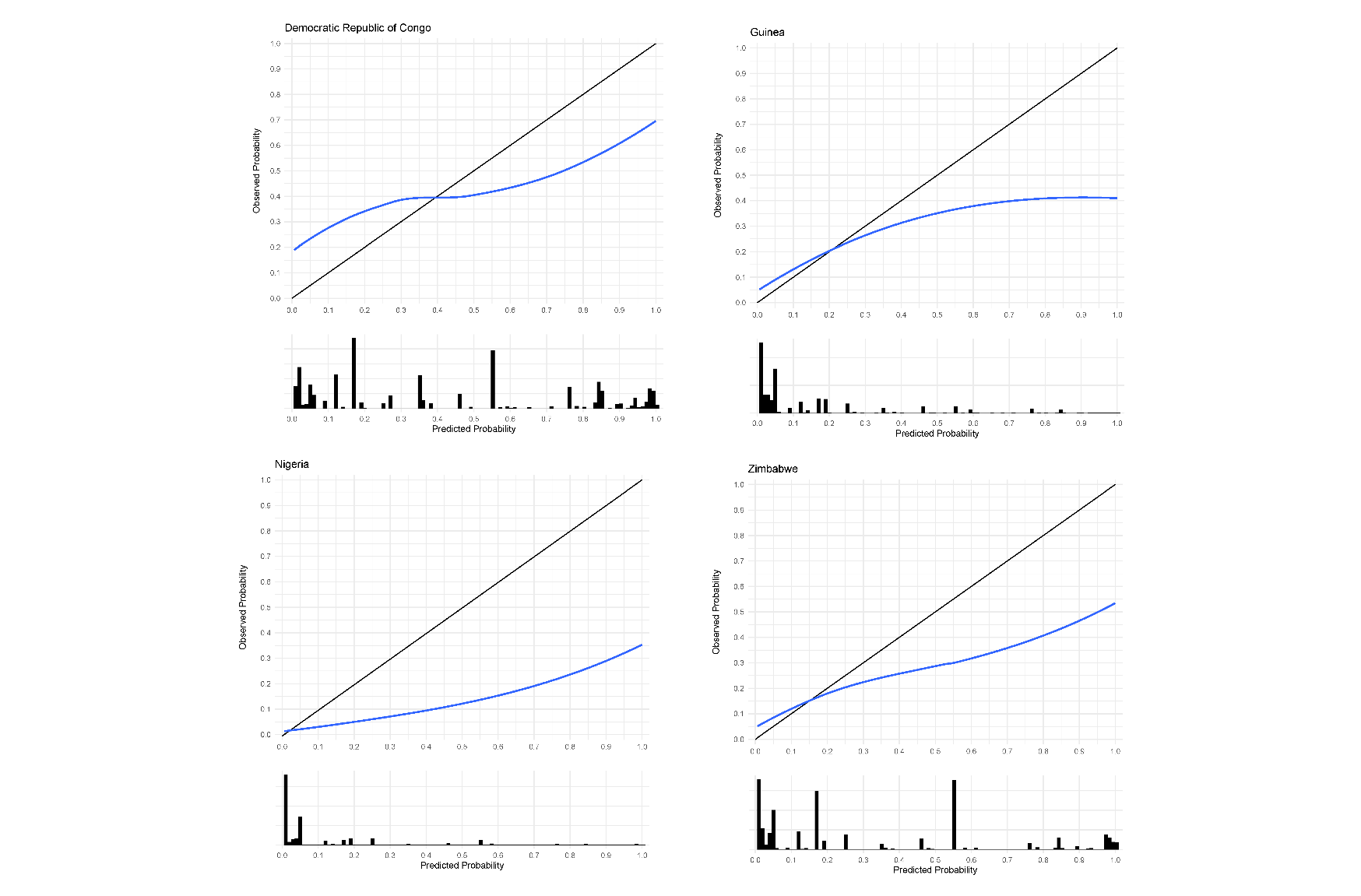


Calibration plots and loess lines were drawn in the stacked dataset (including all 50 imputed datasets) in the total population.
